# In silico analysis of soybean-derived umami peptides: Discovery and interaction mechanisms with T1R1/T1R3 receptor

**DOI:** 10.1016/j.fochx.2025.102544

**Published:** 2025-05-10

**Authors:** Xiaoli Shen, Hao Zhang, Pengyin Zhang, Xiaodi Niu, Xuerui Zhao, Lvzhou Zhu, Jinyang Zhu, Song Wang

**Affiliations:** a Institute of Theoretical Chemistry, College of Chemistry, Jilin University, 2 Liutiao Road, Changchun 130023, PR China; b School of Life Sciences, Jilin University, 2699 Qianjin Street, Changchun 130012, PR China; c College of Food Science and Engineering, Jilin University, Changchun 130062, PR China

**Keywords:** Umami, Virtual enzymatic hydrolysis, Soybean protein, Electronic tongue, Molecular dynamics simulation

## Abstract

In this study, umami peptides with binding activity to the umami receptor T1R1/T1R3 were screened and identified from soybean protein. Using virtual enzymatic hydrolysis, a total of 629 dipeptides to hexapeptides were generated. Through predictions of bioactivity, water solubility, and hemolytic activity, 43 non-toxic peptides were selected. Deep learning methods were employed to predict the umami characteristics of these peptides, ultimately leading to the identification of 17 peptides with potential umami properties. Further molecular docking analysis revealed that the peptides DSWPSL, SHHPR, LGPK and SSW exhibited high binding stability with the umami receptor, indicating strong umami characteristics. The umami properties of these peptides were confirmed through electronic tongue experiments and sensory evaluation, with SHHPR exhibiting the lowest bitterness in sensory evaluation, making it seem more suitable for consumption in food. Molecular dynamics simulations uncovered the interaction mechanisms between the umami peptides and T1R1/T1R3, highlighting charge-charge forces as the primary interaction. This study not only provides new insights for the development of natural umami enhancers but also demonstrates the integration of food science and computational techniques.

## Highlights

• Soybean-derived peptides offer a sustainable source for umami peptide discovery.
• Four deep learning models identified 17 peptides with strong binding to T1R1/T1R3.
• SHHPR showed low bitterness and high umami, making it ideal for flavor enhancement.
• Electrostatic forces dominate peptide binding to T1R1/T1R3, as revealed by molecular dynamics.
• Sensory evaluation confirmed peptides' umami, advancing flavor development.

## 1 Introduction

**Fig. 1:**
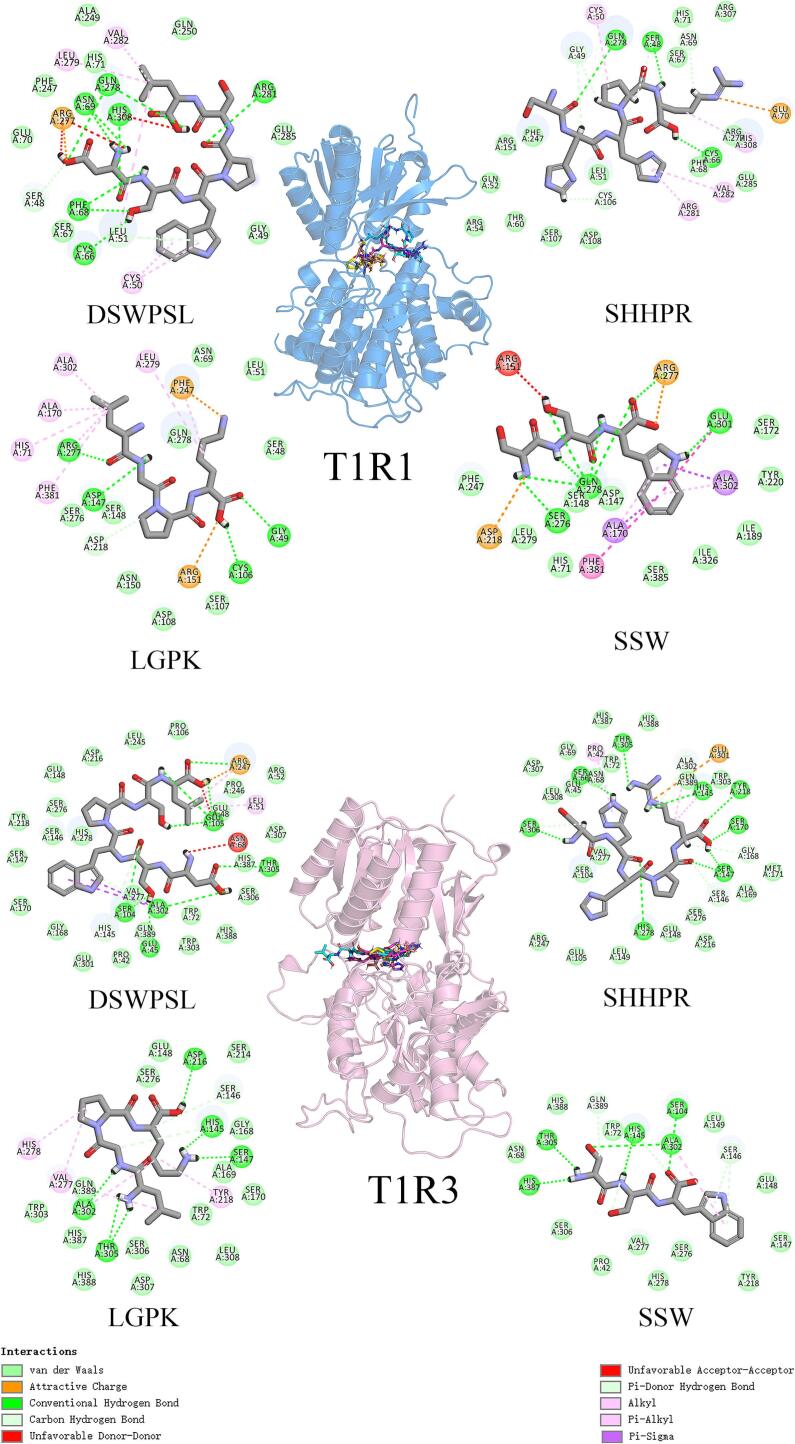
The contact residues and interactions between umami peptides and their binding sites on the protein.

Umami is one of the five basic tastes in humans. Its unique flavor experience not only holds a significant place in cooking and the food industry but is also closely related to the nutritional value and health benefits of food (Magerowski et al., 2018; Sasano et al., 2010; Zhang et al., 2020). With the advancement of modern science and technology, research into the molecular mechanisms of umami formation has progressed significantly. Several studies have pointed out that the perception of umami in humans depends on the function of the T1R1/T1R3 umami receptor (Behrens et al., 2018; Nelson et al., 2002; Zhang et al., 2019; J. Zhao, Liao, et al., 2023). In the field of food science, research on umami receptors helps to understand the mechanisms of umami substance perception in food. This research can guide the optimization of food formulations and the improvement of processing technologies. For instance, by screening and designing small molecules that bind to T1R1/T1R3, foods with specific umami characteristics can be developed to meet the diverse demands of consumers (Chang et al., 2023). Moreover, these studies can assist in evaluating the content and quality of umami substances in food, thus providing a scientific basis for food quality control and safety assessment. In the field of drug development and disease treatment, the umami receptor T1R1/T1R3 also holds significant potential for application (Ahmad & Dalziel, 2020). Some diseases, such as diabetes (Siddiqui et al., 2013) and hypertension (Doty, 2019), are often accompanied by symptoms like taste abnormalities and changes in appetite. By regulating the expression of T1R1/T1R3, it is possible to improve the taste experience and enhance the appetite of these patients. Additionally, the T1R1/T1R3 receptor may also be involved in regulating physiological processes such as energy metabolism and obesity, making them potential innovative drug targets for the prevention and treatment of related diseases (Kochem, 2017; Treesukosol et al., 2011). The T1R1/T1R3 heterodimer belongs to the class C G-protein-coupled receptor (GPCR) family. Its structure consists of a large extracellular N-terminal domain (also known as the Venus Flytrap Domain, VFD), an extracellular cysteine-rich domain (CRD), a 7-transmembrane domain (7-TMD), and an intracellular C-terminal tail domain (Fig. 1). The VFD functions as the ligand-binding module, used to distinguish and recognize taste patterns, while the CRD serves as a bridge between the VFD domain and the 7-transmembrane domain (7-TMD) (Zhang et al., 2019; N. Zhang, Cui, et al., 2022). Both the VFD domains of T1R1 and T1R3 possess a cavity, which is considered a crucial binding site for umami peptides and plays an essential role in the mechanism of umami perception (Dang et al., 2019). This cavity is flanked by α-helices and β-sheets on both sides (Fig. S1).

Umami peptides are a class of small peptides that can activate umami receptors and produce the perception of umami. They trigger umami signals in the human taste system, providing a pleasurable sensory experience (Ghirri & Bignetti, 2012; Shu et al., 2023). At the same time, umami peptides also possess a range of bioactive properties, such as antioxidant and anti-inflammatory effects, which give them broad application prospects in the fields of food, medicine, and health products (Shen et al., 2021; Wang et al., 2024).

Soybeans, as a widely cultivated crop rich in protein, have become a prominent focus in scientific research due to their potential bioactive components (Cai et al., 2021; Hu et al., 2023). Additionally, specific enzymatic hydrolysis or fermentation techniques can be used to extract peptides with umami characteristics from soybeans. For example, Amin et al. employed LC-MS/MS analysis and database-assisted identification techniques to identify a novel umami peptide, GENEEEDSGAIVTVK (GK-15), from the small peptide (<3 kDa) fraction of Danbei water extract (Amin et al., 2020). This method offers several advantages, including high throughput and accuracy, enabling efficient and precise identification of multiple peptides. The use of 3 kDa membrane ultrafiltration effectively isolates small peptides, ensuring high purity for subsequent functional studies. Additionally, database-assisted identification enhances the reliability of the results, while electrospray ionization (ESI) coupled with C18 column separation offers high sensitivity, enabling the detection of low-abundance peptides. The automated data processing also improves experimental efficiency. However, the method has certain limitations. It involves multiple complex steps, making it time-consuming; the equipment is expensive, requiring specialized maintenance, which can be a challenge for low-budget labs; extraction efficiency is limited, as some peptides may not be captured; and the data processing, though comprehensive, can be slow and is dependent on the completeness of the databases and the accuracy of search algorithms. Furthermore, sample matrix interference may cause signal suppression or misidentification. Although LC-MS/MS provides significant advances in peptide identification, its limitations in extraction efficiency, complexity, and cost make it complementary to computational approaches like virtual screening, which offer lower costs and higher efficiency. Despite these drawbacks, this method significantly advances the identification of umami peptides, though it still requires complementary approaches to address its limitations. Consequently, research on soybean umami peptides has increased in recent years, particularly in areas such as extraction processes, structural identification, and functional evaluation (C. Yang, Mu, et al., 2024). Numerous researchers have employed various methods, including optimizing extraction conditions (Duppeti et al., 2023) and utilizing modern analytical techniques (L. Su, Ji, et al., 2024), to continuously improve the extraction efficiency and purity of umami peptides. In response to the challenges associated with traditional methods, virtual screening based on computational techniques has emerged as a promising alternative, offering low-cost, high-efficiency solutions to peptide screening (Kumar & Zhang, 2015; Lin et al., 2020).

Molecular docking has made significant contributions to the screening of peptides with various functions, including angiotensin-converting enzyme (ACE) inhibitory peptides (Guan et al., 2016; Wu et al., 2014), bitter peptides (W. Zhao, Zhang, et al., 2023), and immunomodulatory peptides (He et al., 2021). Molecular docking techniques have been widely applied in the screening of bioactive peptides (Cai et al., 2023; Igbokwe et al., 2024). However, molecular docking cannot thoroughly describe changes in protein conformations, which subsequently leads to limitations in the accuracy of energy predictions. Therefore, it is necessary to combine it with other computational methods.

Through predictive analysis, machine learning can transform vast amounts of data into valuable knowledge and assist decision-making across various applications. In recent years, machine learning has achieved numerous successful research outcomes in fields such as medical diagnosis, drug screening, and toxicity prediction (Cavasotto & Scardino, 2022; Kourou et al., 2021; J. Su, Liu, et al., 2024b; Vatansever et al., 2021). Similarly, it has also been widely used for predicting the molecular characteristics of food.

This study aims to screen umami peptides with T1R1/T1R3 binding activity from soybean protein. We adopted a multi-stage computational strategy, incorporating virtual enzymatic hydrolysis, peptide property prediction, and molecular dynamics simulations, to systematically identify potential umami peptides. First, virtual enzymatic hydrolysis of soybean protein was performed, and the resulting peptide segments were screened for water solubility, hemolytic activity, bioactivity, and toxicity. Next, we utilized four deep learning methods, including WLN, HGNN5, AttentiveFP, and GraphSAGE to predict properties such as the taste of the molecules. Then, the selected peptide segments were docked with T1R1 and T1R3 to identify potential umami peptides. Subsequently, the umami activity of these potential peptides was verified through electronic tongue experiments and sensory evaluations. Finally, molecular dynamics simulations were conducted to reveal the interaction mechanisms between T1R1/T1R3 and the umami peptides. Through this approach, we not only identified a series of novel umami peptides but also provided a more efficient and cost-effective pathway for screening taste molecules. Our work demonstrates the integration of advanced computational methods in food science, paving the way for further research into the development of flavor-enhancing peptides.

## 2 Materials and methods

### 2.1 Materials

The umami peptides were synthesized by Wuhan Dangang Biotechnology Co., Ltd. (Wuhan, China). The purity of the peptides was over 95 %, and their identities were confirmed by mass spectrometry and high-performance liquid chromatography (detailed experimental procedures are available in Supplementary Information).

### 2.2 Virtual screening of peptides

#### 2.2.1 Virtual enzymolysis of protein

The soybean protein sequence was obtained from the National Center for Biotechnology Information (NCBI) and can be accessed via https://www.ncbi.nlm.nih.gov/guide/all/ (accessed on March 13, 2024) (NCBI: KRH47534.1) (Schmutz et al., 2010; Zhao et al., 2019). This sequence was chosen based on previous studies that have extracted sweet peptides from it (J. Su, Liu, et al., 2024b). Since sweet taste and umami receptors belong to the same family, the sequence is highly relevant to our research. Additionally, other studies have used this sequence to identify ACE inhibitors (Ramlal et al., 2022). We used the ExPASy PeptideCutter tool (http://web.expasy.org/peptide_cutter/) (accessed on March 13, 2024) (Gasteiger et al., 2003) to hydrolyze the soybean protein using three typical enzymes. These enzymes include pepsin (pH > 1.3, EC 3.4.23.1) (Li et al., 2018), chymotrypsin (EC 3.4.21.2) (Kurth et al., 1997), and trypsin (EC 3.4.21.4) (Yu et al., 2018; Zhao et al., 2019). The peptides generated from the hydrolysis process were used for subsequent predictive analysis.

#### 2.2.2 Prediction of bioactivity, peptide water solubility, and hemolytic activity

According to studies, short peptides (ranging from dipeptides to tetrapeptides) account for a significant proportion (about 40 %) of known umami peptides, yet only a few hundred have been identified (Zhang et al., 2024). Furthermore, research by Chen et al. also indicates that hexapeptides exhibit higher umami scores (Chen et al., 2023). Therefore, based on these prior findings, we selected dipeptides to hexapeptides for bioactivity prediction after virtual hydrolysis. The predictions were conducted using PeptideRanker (http://peptideep.ucd.ie/PeptideRanker/) (accessed on March 13, 2024), which provides a prediction probability ranging from 0 to 1. Peptides with a score higher than 0.5 were identified as having bioactivity (Lafarga et al., 2015; Mooney et al., 2012). Additionally, to ensure the practical feasibility of these peptide segments, we selected those with higher solubility for further analysis.

First, we used RDKIT to convert the selected peptide sequences into SMILES (Simplified Molecular Input Line Entry System). Then, we employed the peptide property calculator available at https://peptide.bio (accessed on March 13, 2024) to assess the solubility and hemolytic risk of the hydrolyzed peptides. Ultimately, we filtered for peptide segments with water solubility greater than 60 % and hemolytic risk below 10 % (Barrett & White, 2021; Pirtskhalava et al., 2021; Smialowski et al., 2012). Hemolytic risk prediction served as an initial screening step in the toxicity assessment, prioritizing the exclusion of peptides with high hemolytic activity and potential blood system toxicity. This approach ensures that subsequent toxicity analysis focuses on candidate peptides with higher biosafety potential. These selected peptides were then prepared for further umami and toxicity predictions.

#### 2.2.3 Prediction of umami and toxicity of peptides

Building on the peptides selected from the previous step, we applied four deep learning methods (including WLN (Morris et al., 2019), HGNN5 (He et al., 2024), AttentiveFP (Xiong et al., 2020), and GraphSAGE (Hamilton et al., 2017)) for umami prediction. These methods can be accessed through the taste-prediction website (https://www.tastepd.com/predict) (accessed on March 15, 2024). The website has been successfully used to predict sweet peptides (J. Su et al., Su, Liu, et al., 2024a), which is why we also chose it for umami prediction. All four deep learning methods rely on Graph Neural Networks (GNN) and their variants for prediction. In these approaches, the atoms in the molecule are treated as nodes in the graph, and the bonds are treated as edges. By using Graph Neural Networks, we can learn the complex relationships between these structures to predict properties, such as the taste of the molecules. By combining the prediction results of these methods, we identified a set of potential umami peptides.

Finally, we performed toxicity screening on these peptides using ADMET Lab 2.0 (Dong et al., 2018; Jiang et al., 2020; Xiong et al., 2021), retaining those predicted to be non-toxic.

This comprehensive evaluation process allowed us to select peptides with high bioactivity, low hemolytic risk, and non-toxicity, enhancing their suitability for subsequent research or product development.

### 2.3 Molecular docking screening

First, the amino acid sequences of the umami receptors T1R1 and T1R3 were retrieved from UniProt (T1R1, UniProtKB: Q7RTX1; T1R3, UniProtKB: Q7RTX0) (Dang et al., 2019). Subsequently, the complete structures of T1R1 and T1R3 were constructed using Alphafold2 (Jumper et al., 2021; Varadi et al., 2024). Finally, molecular docking was performed using Autodock Vina 1.2.0 (Eberhardt et al., 2021; Forli et al., 2016). For docking with T1R1, the docking box was centered around Glu70 and Gln278, with dimensions set to x = 45.15, y = 40.85, and z = 45.15. For docking with T1R3, Glu45, Glu148, and His278 were used as boundaries for the docking box, which was set to x = 22.5, y = 18.75, and z = 18.75, with a grid spacing of 0.0375 nm. Four peptides were docked into the cavities of the two proteins, and after docking each system, nine docking results were generated, with the conformation having the lowest binding energy selected for further study.

### 2.4 Taste analysis by electronic tongue (E-tongue)

In this study, we employed the SA402B electronic tongue from Insent, Atsugi City, Japan, for taste analysis. This device utilizes a complex system of artificial lipid membrane sensors to detect a range of tastes. Specifically, the C00 sensor detects acidic bitterness with an aftertaste of acidic bitterness (Aftertaste-B), the AE1 sensor detects astringency with an aftertaste of astringency (Aftertaste-A); the CA0 sensor detects sourness with no aftertaste; the CT0 sensor detects saltiness with no aftertaste; and the AAE sensor detects umami with an aftertaste of richness.

Our test solution consisted of 30 mM potassium chloride and 0.3 mM tartaric acid. Two different cleaning solutions were prepared for the electrodes: the cleaning solution for the negative electrode contained 100 mM hydrochloric acid mixed with 30 % ethanol by volume, while the cleaning solution for the positive electrode consisted of 10 mM potassium hydroxide, 100 mM potassium chloride, and 30 % ethanol by volume. The concentration of the sample solution was set to 0.1 mg/mL, and the pH of the umami peptide solution was in the range of 6–7 (F. Yang, Meng, et al., 2024). The testing protocol for the SA402B electronic tongue involved a multi-step process. First, the sensors were cleaned in their respective solutions for 90 s, followed by a 120-s cleaning in the reference solution, which was repeated twice. Next, the sensors were calibrated to zero at equilibrium for 30 s. During the testing phase, each test lasted 30 s, during which the instantaneous taste values were recorded. After testing, the sensors were quickly cleaned in the reference solution for 3 s and then inserted into a new reference solution for 30 s to assess the aftertaste. The taste sensors (C00, AE1, CA0, CT0, AAE) underwent four cycles of this testing procedure. The results from the first cycle of each test were discarded, and the final results were based on the average of the three subsequent cycles. This rigorous methodology ensured reliable and accurate assessments of the taste characteristics of the samples.

### 2.5 Sensory evaluation

The experimental panel consisted of 10 participants (5 females and 5 males, aged 25–45), all of whom had no smoking or excessive drinking habits. Prior to the sensory evaluation, all panel members underwent a screening and training process. During the screening test, the participants were asked to assess the intensity of five basic taste solutions. The standard solutions for the five basic tastes were prepared as follows: umami—MSG solution (3.5 mg/mL), sweet—sucrose solution (10 mg/mL), salty—sodium chloride solution (3.5 mg/mL), bitter—L-isoleucine solution (2.5 mg/mL), and sour—citric acid solution (0.8 mg/mL). During the training, the panel members practiced repeatedly to familiarize themselves with the sensory perception of each solution and learned how to accurately record sensory descriptions to ensure consistency in the evaluation. After the training, all panel members were required to pass a screening test, which involved assessing a set of reference solutions to confirm that they could reliably evaluate taste intensity and accurately identify the five basic tastes. Only those who passed the screening test were eligible for the formal sensory evaluation experiment (Shan et al., 2022). The sensory evaluation was conducted in a professional sensory analysis laboratory. The temperature in the laboratory was strictly controlled at (22.5 ± 2.5) °C under normal lighting conditions. All synthetic peptide solutions used in the sensory evaluation were prepared at a concentration of 2 mM, and the pH of the umami peptide solution was maintained in the range of 6–7 using 1 M citric acid or 1 M NaOH. During the evaluation, panel members used a 0 to 5-point scale to assess the taste intensity of each sample, where 0 indicated “undetectable” and 5 indicated “strongly detectable.” The standard solution was assigned a score of 2.5, which served as a baseline for evaluating the taste intensity of other samples.

Samples were assigned four-digit random codes. Panel members were instructed to swirl the samples in their mouths for 10 s before expectorating. After each tasting, they rinsed their mouths with ultrapure water (50 mL) at least twice and rested for 2 min to prevent taste fatigue and residual effects. The sensory evaluation of each sample was conducted three times. All experimental procedures adhered to the ethical principles of the World Medical Association regarding human experimentation (Helsinki Declaration).

### 2.6 Molecular dynamics simulation

To investigate the binding modes and mechanisms of the selected umami peptides with umami receptors, we conducted molecular dynamics (MD) simulations on their complexes. The study revealed that the VFD regions of T1R1 and T1R3 are the primary ligand binding domains (Li, 2009; Zhang et al., 2008). Consequently, after performing molecular docking, we used the VFD domains of T1R1 and T1R3, along with the ligands, as complexes for the MD simulations. We utilized the pmemd.cuda module of AMBER 22 (Case et al., 2021) to perform MD simulations on all T1R1/T1R3-peptide complexes obtained through screening. For the parameterization of proteins, peptides, and water molecules, we employed the ff14SB force field from AMBER 22 (Maier et al., 2015) and the TIP3P water model (Jorgensen et al., 1983) were used. The latter was also employed to create octahedral water boxes around each system. The distance between the water box and the surface of the solute was set to 0.8 nm and periodic boundary conditions were applied to mitigate edge effects. Subsequently, sodium and chloride ions were added to the T1R1-peptides and T1R3-peptides systems to neutralize the systems.

Before the simulation, we first conducted energy minimization with 500 steps each of the steepest descent and conjugate gradient algorithms to eliminate atomic clashes. Next, under the NVT ensemble, the system was gradually heated from 0 K to 300 K over a period of 50 ps. Following this, a 50 ps NPT ensemble equilibration was performed to ensure that the system was in a stable environment. Once the thermodynamic properties of the system stabilized, we conducted a 200 ns MD simulation. The Particle-Mesh Ewald (PME) method (Darden et al., 1993) was employed to manage electrostatic interactions, utilizing a cutoff distance of 0.8 nm. Hydrogen bonds were constrained using the SHAKE algorithm (Hopkins et al., 2015). The time step for integration was set to 2 fs, and a Langevin thermostat with a collision frequency of 1 ps was employed to maintain a constant temperature in the system (Davidchack et al., 2009).

## 3 Results and discussion

### 3.1 Virtual screening of peptides

Through virtual enzymatic digestion, a total of 629 dipeptides to hexapeptides were generated from the hydrolysate of soybean protein. Based on bioactivity predictions –where a score above 0.5 in PeptideRanker was considered bioactive–118 peptides were identified as bioactive, as shown in Table S1. Among these 118 bioactive peptides, 43 exhibited good water solubility and low hemolytic activity, with further details provided in Table S2. All peptides were predicted to be non-toxic. Subsequently, four deep learning models–WLN, HGNN, AttentiveFP, and GraphSAGE– were employed to predict the umami taste of these 43 peptides. The prediction results are shown in Table S3. Of these, 17 peptides were identified by at least three of the models as having an umami taste (Table 1).

**Table 1:** Umami prediction results of peptides.

Peptide	Number of amino acid	Soluble	Hemolysis	Peptide ranker	Toxicity
DSWPSL	6	90 %	0 %	0.785783	Non-toxic
SSW	3	98 %	1 %	0.731445	Non-toxic
CS	2	98 %	1 %	0.513886	Non-toxic
SSAMM	5	97 %	1 %	0.686530	Non-toxic
GDMDY	5	96 %	1 %	0.589397	Non-toxic
APAP	4	93 %	1 %	0.700620	Non-toxic
AMQL	4	91 %	1 %	0.545898	Non-toxic
SHHPR	5	91 %	1 %	0.538536	Non-toxic
GGL	3	68 %	1 %	0.838635	Non-toxic
GL	2	61 %	1 %	0.808777	Non-toxic
SC	2	98 %	2 %	0.620334	Non-toxic
SAC	3	97 %	2 %	0.503776	Non-toxic
AM	2	86 %	2 %	0.745490	Non-toxic
CPK	3	94 %	3 %	0.648049	Non-toxic
GLPK	4	93 %	3 %	0.561671	Non-toxic
SW	2	98 %	5 %	0.933910	Non-toxic
LGPK	4	72 %	5 %	0.516678	Non-toxic

### 3.2 Potential umami peptide screening

To further screen for umami peptides, we first validated the reasonableness of the T1R1 and T1R3 homology models, with their Ramachandran plots shown in Fig. S2. According to the 90 % criterion evaluation principle, the two models were considered reasonable in terms of dihedral angle distribution and spatial clashes. Subsequently, we docked 17 selected peptides with T1R1 and T1R3 to evaluate their interactions with the receptor subunits. The absolute values of the docking scores were used as indicators of the binding stability and strength between the receptor and the ligand (Table 2). Higher absolute values indicate tighter binding to the receptor, with the peptides DSWPSL, SHHPR, LGPK and SSW demonstrating stronger affinities for the umami receptors, suggesting they may have umami flavor. Further analysis revealed that the interactions between these peptides and T1R1/T1R3 were primarily driven by van der Waals forces and hydrogen bonds (Fig. 1). A total of 28 residues in the receptors participated in the interactions with these peptides. In the case of T1R1, the residue Gln278 was involved in the majority of the ligand-receptor interactions, a finding consistent with previous studies (Wang et al., 2022; C. Zhang, Miao, et al., 2022). In contrast, the residues Ala302, Thr305, Ser147, and His145 in T1R3 showed significant interactions with all peptides, which is also in line with earlier research (Gu et al., 2025). Moving forward, these peptides will be the main targets for further synthesis and investigation of their mechanisms of action with the receptors.

**Table 2:** Molecular docking energy of the predicted umami peptides (kcal/mol).

Peptide	T1R1	T1R3	Sum
DSWPSL	−7.5	−9.7	−17.2
SHHPR	−7.5	−9.1	−16.6
LGPK	−7.9	−7.8	−15.7
SSW	−8	−7.6	−15.6
APAP	−7.3	−8.2	−15.5
GDMDY	−7.4	−8.1	−15.5
AMQL	−7.2	−7.7	−14.9
SW	−7.4	−7.2	−14.6
SSAMM	−7.3	−7	−14.3
GLPK	−6.2	−8	−14.2
CPK	−6.5	−6.8	−13.3
SAC	−5.9	−6.4	−12.3
GGL	−5.7	−6.3	−12
GL	−5.9	−5.6	−11.5
CS	−5.6	−5.6	−11.2
AM	−5.7	−5.3	−11
SC	−5.6	−5.2	−10.8

### 3.3 Electronic tongue analysis and sensory evaluation of synthesized peptides

**Fig. 2:**
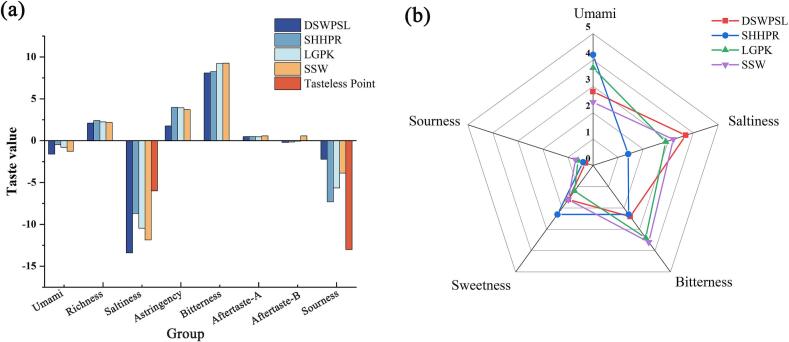
The taste profile curves of the synthesized peptides. (a) Electronic tongue test results. (b) Sensory evaluation results.

Before starting the experiments, we verified the purity of the four selected peptides using high-performance Liquid Chromatography–Mass Spectrometry (HPLC–MS) (see Supplementary Information for the detailed experimental procedures). The results indicated that the purity of each peptide reached 95 %, as presented in Fig. S3. Additionally, we performed TIC and MS/MS analysis to further confirm the identity and purity of the peptides. The TIC plots and MS/MS spectra are shown in Fig. S4, where distinct peaks and fragmentation patterns validate the structural integrity of the peptides. Subsequently, we conducted electronic tongue experiments and sensory evaluations, with the results illustrated in Fig. 2 (the electronic tongue results are displayed as bar charts, while the sensory evaluation results are shown as radar charts).

In the electronic tongue experiment, a baseline was established using the neutral point (the output of the reference solution). The baseline taste values were determined based on the reference solutions of KCl and tartaric acid: the neutral point for sourness was −13, and for saltiness, it was −6. When the taste value of a sample is lower than the neutral point, the sample does not possess that flavor; a higher value indicates the presence of that flavor. The electronic tongue experiment did not detect a clear umami taste for the four peptides, but it did detect a higher richness. Richness refers to the aftertaste of umami, reflecting the persistence of umami flavor in the sample, also known as umami durability.

It is important to note that the results of the electronic tongue experiments are primarily based on the electrode response values (Latha & Lakshmi, 2012). The release of umami components is largely dependent on the oral processing, with saliva playing a crucial role in releasing these components (Chen, 2014). Therefore, we also conducted sensory evaluation experiments. The results indicated that all participants detected umami flavor in the four peptides, with the intensity of umami ranked as follows: SHHPR > LGPK > DSWPSL > SSW. In the electronic tongue experiment, distinct umami was not detected; instead, all four peptides were found to have richness. Richness here refers to the fullness, depth, and duration of umami perceived in the mouth. The electronic tongue sensors cannot precisely describe the release process of umami in the mouth, while sensory evaluation is susceptible to individual differences and external environmental factors (Li et al., 2023). Furthermore, if the team members have not received professional training, there will be differences in the description and evaluation of taste (Tan & Xu, 2020). Therefore, combining the electronic tongue with sensory evaluation to analyze the effects of umami can enhance the reliability of the data.

Moreover, the differences between the electronic tongue experiment and sensory evaluation may be due to differences in solution concentrations. In this study, the peptide solution concentration used in the electronic tongue experiment was 0.1 mg/mL, whereas the concentration used in the sensory evaluation was 2 mM. This concentration difference could affect the perception of umami intensity. Specifically, the low concentration in the electronic tongue experiment may result in a weaker sensor response, making it difficult to detect distinct umami components, primarily reflecting the “richness” of the umami-i.e., its fullness, depth, and persistence. The sensitivity of the electronic tongue generally depends on the concentration of the target substance in the solution, and at lower concentrations, it may fail to reach the detection threshold, leading to the omission of umami signals. In contrast, the 2 mM concentration used in the sensory evaluation makes it easier for umami components to bind to taste receptors, allowing participants to perceive umami more effectively. Therefore, the difference between the electronic tongue and sensory evaluation results may stem from the effect of concentration on taste perception. Additionally, electronic tongue devices cannot fully replicate the complex interactions of taste in the mouth, particularly for umami, which is significantly influenced by factors such as saliva, temperature, and mastication. On the other hand, sensory evaluation can capture a more complex and comprehensive taste experience through human sensory responses. Taking these factors into account, all four peptides were identified as umami peptides, with SHHPR showing the lowest bitterness in sensory evaluation, making it more suitable for use in food applications.

### 3.4 Mechanism of interaction between peptides and T1R1, T1R3

The previous experimental results indicated that all four peptides possess umami characteristics. Therefore, we further employed MD simulations to investigate the dynamic stability and binding free energy of eight complexes formed by these four umami peptides with the T1R1 and T1R3 subunit proteins (named T1R1-DSWPSL, T1R1-SHHPR, T1R1-LGPK, T1R1-SSW, T1R3-DSWPSL, T1R3-SHHPR, T1R3-LGPK, and T1R3-SSW), aiming to explore their umami mechanisms in depth.

**Fig. 3:**
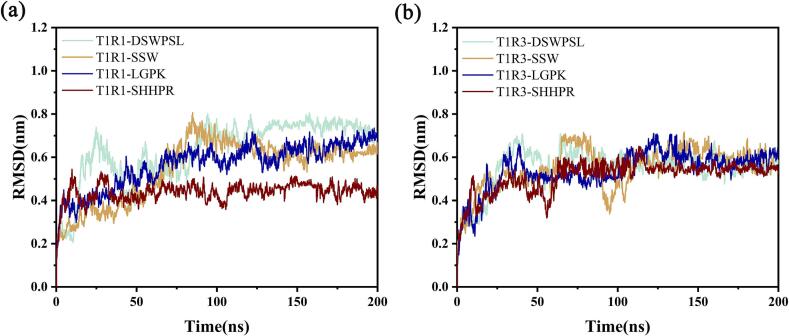
The RMSD-simulation time curves of the complex systems. (a) T1R1-peptide. (b) T1R3-peptides.

To assess the dynamic stability of the T1R1 and T1R3-peptide complexes, we conducted root mean square deviation (RMSD) analysis. The RMSD values reveal the deviation of the complexes from their initial conformations, reflecting the extent to which the protein molecules diverge from their initial structure during the MD simulation. As shown in Fig. 3, for the T1R1-peptide complexes, the curves for T1R1-DSWPSL and T1R1-SSW exhibited significant fluctuations during the first 100 ns, after which the system stabilized with increasing simulation time. In contrast, the curves for T1R1-SHHPR and T1R1-LGPK remained relatively stable throughout the entire simulation. Similarly, in the T1R3-peptide complexes, T1R3-LGPK and T1R3-SHHPR demonstrated more stable interactions with the protein. This suggests that complexes formed with LGPK and SHHPR are more likely to reach dynamic equilibrium and stability, indicating that these peptides may bind more effectively to the receptor. These observations provide valuable insights for subsequent computational studies.

To further quantify the interactions between peptides and their receptors, the Molecular Mechanics Poisson-Boltzmann Surface Area (MM-PBSA) method was employed to calculate the binding free energies of the eight complexes formed between the four peptides and the two receptor subunits, T1R1 and T1R3. The analysis utilized molecular dynamics (MD) simulation trajectories from the initial 100 ns production phase. Although we monitored the subsequent 100 ns trajectory for system stability, no significant ligand displacement was observed beyond the initial 100 ns period. Therefore, the first 100 ns were selected for energy calculations. For each trajectory, conformational sampling was performed by extracting 20 equally spaced frames at 5 ns intervals. Table 3, Table 4 present the binding free energies for the peptides with T1R1 and T1R3, respectively. The contributions to the total binding free energy (ΔG_total_) are broken down into three key components: the van der Waals contribution (ΔG_vdw_), the electrostatic contribution (ΔG_elec_) and the solvation energy contribution (ΔG_PB_). The total free energy, ΔG_total_, is calculated as the sum of these components, providing a comprehensive view of the interaction stability between each peptide and its respective receptor subunit. This data enhances the understanding of the molecular interactions that drive the binding affinity and stability of the peptides with umami taste receptors. It is important to note that in both the T1R1-peptide and T1R3-peptide systems, the electrostatic contribution is significantly greater than the van der Waals contribution. This suggests that electrostatics may play a primary role in binding interactions. This phenomenon may be attributed to the charged residues located at both ends of the peptide, which can establish strong charge-charge interactions with residues in the protein that possess charged side chains. Notably, SHHPR exhibits the largest |ΔGelec| with the T1R3 subunit, reaching 313.8844 kcal/mol. This may be due to the presence of an additional charged arginine, in addition to the charged residues at both ends. However, the number of residues with charged side chains is not the absolute factor affecting affinity. For example, DSWPSL contains an Aspartic acid with a charged side chain, while SSW has no residues with charged side chains. In the T1R1-peptide system, the ΔG_total_ values for both peptides are nearly the same. Additionally, both SHHPR and LGPK contain a positively charged residue, yet their ΔG_total_ values differ significantly within the same protein. This highlights the importance of peptide ligand binding positions within the protein, with a more detailed discussion of binding free energy from residue decomposition to be presented in the next section. Moreover, longer peptides like DSWPSL and SHHPR, which have nonpolar side chains (especially aromatic side chains), show stronger van der Waals contributions compared to the other peptides, suggesting that nonpolar interactions also play a significant role in the protein-peptide binding. In contrast to the direct interactions between the protein and the peptide, the polar ΔG_PB_ from the solvent negatively impacts the binding of the two, and this effect is inversely related to the electrostatic interactions; as the negative value of ΔG_elec_ increases, the positive value of ΔG_PB_ also increases. If we consider the binding free energy of the peptide to the protein (taking into account only the direct interactions of ΔG_elec_ + ΔG_vdw_) to be positively correlated with umami, then the order of binding free energy for different peptides with T1R3 aligns more cosely with experimental results. Overall, T1R3 shows a higher affinity for the tested peptides, suggesting its predominant role in umami perception. Furthermore, if charged residues are present in the peptide, the ligand is more likely to form electrostatic interactions with the umami receptor.

**Table 3:** Binding free energy of peptides with T1R1 (kcal/mol).

Energy Contribution	T1R1-DSWPSL	T1R1-SHHPR	T1R1-LGPK	T1R1-SSW
ΔG_vdw_	−38.5029	−54.4343	−31.7295	−19.2272
ΔG_elec_	−160.1960	−117.5802	−43.6824	−150.4236
ΔG_PB_	184.7778	139.2912	70.7131	151.5143
ΔG_total_	−13.9211	−32.7233	−4.6988	−18.1365

**Table 4:** Binding free energy of peptides with T1R3 (kcal/mol).

Energy Contribution	T1R3-DSWPSL	T1R3-SHHPR	T1R3-LGPK	T1R3-SSW
ΔG_vdw_	−60.5861	−50.1528	−34.2336	−31.9470
ΔG_elec_	−123.7566	−313.8844	−159.0918	−80.3088
ΔG_vdw_ + ΔG_elec_	−184.35	−364.03	−193.32	−112.26
ΔG_PB_	163.1997	330.5635	183.0386	115.5252
ΔG_total_	−21.1430	−33.4738	−10.2868	3.2695

The dissociation free energy provides a clearer understanding of the binding interactions between different residues and the ligands. Table 5, Table 6 present the key residues of interest within the T1R1-peptide and T1R3-peptide systems, while other residues that significantly contribute to the binding energy are detailed in Tables S4 and S5. In this context, negative values indicate electrostatic attraction, whereas positive values denote repulsion. From these tables, it can be observed that the residues with charged side chains play a crucial role in ligand binding for both T1R1 and T1R3.

**Table 5:** The ΔG_elec_ contributions of key residues in the T1R1-peptide system (kcal/mol).

	T1R1-DSWPSL	T1R1-SHHPR	T1R1-LGPK	T1R1-SSW
Arg54	−14.5651	6.7623	10.1322	0.7476
Arg56	−7.9003	6.6032	5.9552	0.3833
Arg64	−12.9431	−15.3552	10.5823	−1.3116
Glu70	4.4561	−13.2863	−7.575	5.6723
Asp108	13.2402	−26.4404	−11.9435	−3.5992
Asp147	12.8453	−24.6416	−24.3789	−9.8022
Arg151	−15.2936	22.8664	10.7209	3.9786
Asp218	12.9051	−28.9935	−17.4318	−18.4904
Asp219	9.3013	−18.3167	−16.9929	−12.9787
Asp253	12.3271	−30.9161	−7.8658	−0.2703
Glu254	7.9506	−9.1721	−5.9315	−0.0942
Arg255	−11.9882	10.8256	8.2277	0.7244
Arg277	−32.9888	18.9323	5.913	−26.398
Arg281	−16.4442	12.1827	8.513	−3.573

**Table 6:** The ΔG_elec_ contributions of key residues in the T1R3-peptide system (kcal/mol).

	T1R3-DSWPSL	T1R3-SHHPR	T1R3-LGPK	T1R3-SSW
Ser104	−2.0155	−4.4881	0.1798	−5.0917
His145	−3.317	3.3493	0.3742	−5.8024
Glu148	18.5326	−16.7274	−5.1271	10.5843
Glu172	6.9224	−7.151	−9.0145	0.112
Asp190	8.0732	−25.8681	−21.1144	−1.8856
Glu301	12.4413	−32.5191	−37.05	−23.6511
Ala302	0.8128	0.5989	0.0831	−6.5617
Asp307	3.2847	−9.8073	−16.6467	−8.0932

**Fig. 4:**
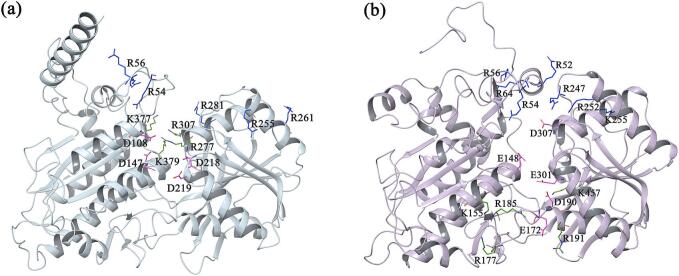
The charge distribution in the cavity regions of (a) T1R1 and (b) T1R3. The blue stick structures represent the residues forming PA1, the purple stick structures represent the residues forming NA1, and the green stick structures represent the residues forming PA2. (For interpretation of the references to colour in this figure legend, the reader is referred to the web version of this article.)

To further analyze the findings, we first conducted a statistical assessment of the charge distribution in the cavities of the two subunit proteins. According to the charge distribution within the protein cavities (Fig. 4), there was a significant accumulation of positive charges at the entrances of the cavities in T1R1 and T1R3 (PA1), while a negative charge-rich region was located deeper within the cavity (NA1). On the opposite side of the negative region, there was also another positive charge-rich area (PA2). The primary difference between the two subtypes was that the NA1 region in T1R1 was relatively shallower. This characteristic led the peptides to favor inserting their positively charged N-terminus and/or side chains deeper into the cavity to match the NA1 region.

**Fig. 5:**
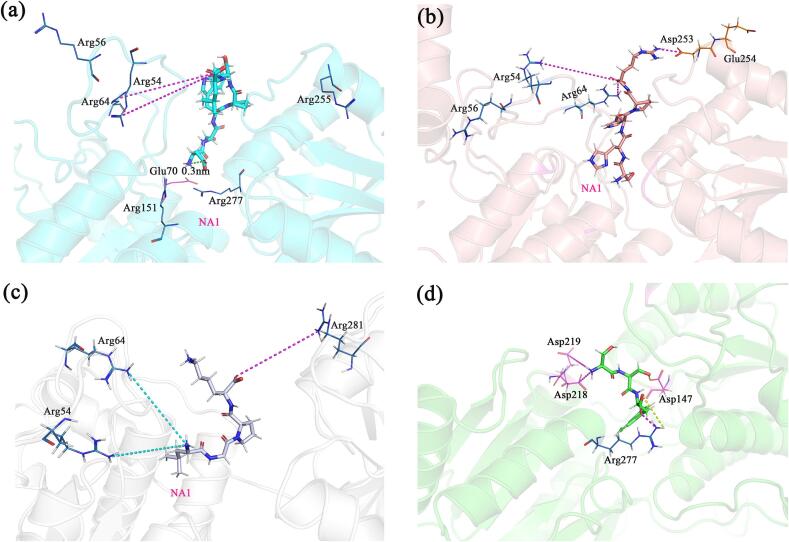
The interaction diagrams of T1R1 with (a) DSWPSL, (b) SHHPR, (c) LGPK, and (d) SSW. The green lines represent the distance between atoms, the purple lines indicate electrostatic attraction, the blue lines indicate electrostatic repulsion, and the yellow lines represent the hydrogen bonds formed. (For interpretation of the references to colour in this figure legend, the reader is referred to the web version of this article.)

In the T1R1-peptide system, the NA1 region consists of Asp108, Asp147, Asp218, and Asp219. The peptides DSWPSL, SHHPR, and LGPK all insert their N-termini into the cavity to align with NA1. However, due to its shorter length, SSW is fully accommodated within the cavity. Notably, DSWPSL features an Aspartic acid at the N-terminus, which possesses a negatively charged side chain that is in close proximity to the terminal NH_4_^+^ group (Fig. 5a). This proximity results in a strong electrostatic repulsion with the negatively charged pocket, preventing the N-terminus of DSWPSL from reaching NA1. Instead, it inserts between the salt bridge formed by Glu70 and Arg277, where the positive and negative charges are complementary, resulting in a very strong interaction.

Additionally, the negatively charged C-terminus of DSWPSL is located at the entrance of the cavity (PA1), where it interacts strongly with surrounding positively charged residues, including Arg54, Arg56, Arg64, Arg151, and Arg255. This interaction significantly enhances the overall electrostatic contribution of DSWPSL to the protein.

Both SHHPR and LGPK have their N-termini entering the NA1 region, with positively charged side chains at their C-termini. However, SHHPR features a longer backbone, which enables its negatively charged C-terminal carboxyl group to interact with Arg54, Arg56, and Arg64 on one side of PA1. Furthermore, its positively charged side chain can engage with the more exterior residues Asp253 and Glu254 (Fig. 5b). In contrast, LGPK has a shorter backbone, and its binding conformation differs somewhat from that of SHHPR. Consequently, the C-terminal carboxyl group of LGPK can interact with Arg281. However, its positively charged side chain is unable to reach the Asp253 and Glu254 region and instead is positioned close to Arg54 and Arg64, resulting in electrostatic repulsion (Fig. 5c). As a result, the overall electrostatic interactions of LGPK are weaker than those of SHHPR. SSW does not carry a net charge, resulting in significantly reduced interactions with the charged residues in the protein. The attractive and repulsive forces from more distant residues are diminished, nearly canceling each other out. Only the strong attraction between the N-terminal -NH_4_^+^ and Asp218/Asp219 in the NA1, as well as between the C-terminal -COO^−^ and Arg277 in the PA2, remains substantial and cannot be neutralized (Fig. 5d). These specific electrostatic interactions are more pronounced than those in other complexes, making them less susceptible to cancellation. This may explain why the electrostatic interactions in SSW are only slightly weaker than those in DSWPSL. Furthermore, the hydrogen bonding interactions in this complex are particularly significant. The statistical analysis of hydrogen bonds across all simulation frames reveals that SSW forms hydrogen bonds with Arg277 and Asp147 in a greater proportion of frames (91 % and 85 % of the total frames, respectively) (Fig. 5d), contributing to its strong binding free energy.

**Fig. 6:**
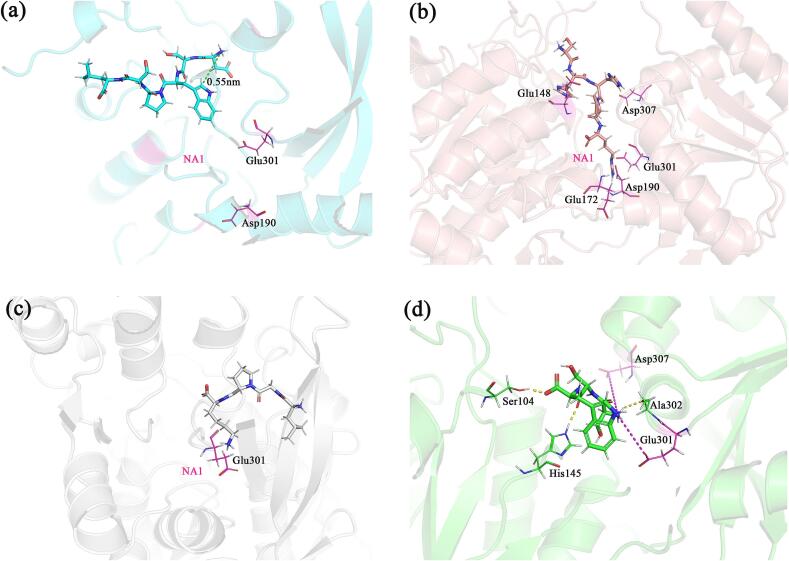
The interaction diagrams of T1R3 with (a) DSWPSL, (b) SHHPR, (c) LGPK, and (d) SSW. The green lines represent the distance between atoms, the purple lines indicate electrostatic attraction, the blue lines indicate electrostatic repulsion, and the yellow lines represent the hydrogen bonds formed. (For interpretation of the references to colour in this figure legend, the reader is referred to the web version of this article.)

In the T1R3-peptide system, the NA1 region is primarily composed of Asp190 and Glu301, with additional contributions from surrounding residues, including Glu148, Glu172, and Asp307. This configuration enables peptides with positively charged N-termini and/or side chains to readily enter NA1 and form charge-charge interactions. However, unlike in T1R1, NA1 in T1R3 is positioned deeper within the cavity and is more constricted. As a result, peptides with large hydrophobic residues near their positively charged groups face steric hindrance, making it difficult for them to access the pocket. When a peptide successfully enters NA1, it forms stronger interactions than in T1R1 due to increased surface contact with the protein; otherwise, the interaction weakens. For instance, in DSWPSL, the Trp3 residue is spatially close to the positively charged N-terminal group and lacks other positive groups, which prevents it from accessing NA1 (Fig. 6a). Consequently, the electrostatic interaction in T1R3 is weaker compared to that in T1R1. In contrast, the C-terminus of SHHPR is able to access NA1, leading to a significant enhancement of its electrostatic interaction compared to T1R1 (Fig. 6b). Similarly, while the N-terminus of LGPK contains a large hydrophobic Leucine side chain that obstructs its entry into NA1, the positively charged Lys4 side chain can still penetrate NA1 (Fig. 6c), resulting in stronger electrostatic interactions than those observed in T1R1.

The binding free energy of SSW with T1R3 is comparable to that with T1R1. However, the charged side chains of the protein contribute relatively weak binding free energy compared to other peptides. Only Glu301 and Asp307 exhibit significant electrostatic attraction; however, their binding free energy is considerably lower than that of the three residues involved in electrostatic interactions in T1R1 due to their spatial arrangement. This discrepancy is likely the primary reason for the notably weaker electrostatic interactions between SSW and T1R3. Additionally, hydrogen bond interactions with residues such as Ser104, His145, and Ala302 are more pronounced (Fig. 6d).

## 4 Discussion

### 4.1 Independent study of T1R1 and T1R3 receptors vs. T1R1/T1R3 heterodimer comparison

In previous studies, molecular docking and molecular dynamics simulations of umami peptides have typically focused on the T1R1/T1R3 heterodimer as the research target (Wang et al., 2022). Although this approach captures the synergistic effects between the two subunits, it may obscure the unique roles of each receptor subunit in umami perception. Moreover, separate studies have reported important roles of T1R1 and T1R3 in umami sensing (Dang et al., 2014; C. Zhang, Miao, et al., 2022). Therefore, in this study, we independently investigated T1R1 and T1R3 to explore the primary subunit responsible for umami perception.

The results showed that the binding free energy ranking of the different ligands with T1R3 was more consistent with the experimental data, whereas the computational results for T1R1 exhibited lower concordance with the experimental findings. Thus, we speculated that umami peptides are more likely to trigger umami perception through interactions with T1R3. SHHPR, due to its C-terminus entering the NA1 region of T1R3, forms more contacts with the receptor, resulting in stronger interactions with T1R3 than with T1R1. In contrast, the large side chain of DSWPSL prevents it from accessing the NA1 region. Although LGPK possesses a bulky hydrophobic leucine side chain, the positive charge at its Lys4 residue enables it to enter the NA1 region, leading to a stronger binding with T1R3 than with T1R1.

Additionally, considering that the four peptides studied contain charged groups, their primary interaction with T1R1/T1R3 is electrostatic. Variations in the types and structures of different umami substances may result in differences in the dominant receptor subunit and key interaction forces when binding to the T1R1/T1R3 heterodimer. This suggests that, during the study of umami perception, different peptides and their structural characteristics may influence the binding modes of T1R1 and T1R3, thereby leading to distinct perception mechanisms.

By independently investigating T1R1 and T1R3, we aim to better understand the distinct contributions of each receptor subunit in umami perception, providing new insights compared to the T1R1/T1R3 heterodimer model, which may obscure these individual roles.

### 4.2 Exploring the discrepancy between strong binding energy and poor umami perception

In this study, we observed that some peptides exhibit high binding free energies with T1R3, yet the corresponding umami perception in sensory experiments does not always align with these findings. For example, while the binding free energy of the peptide DSWPSL with T1R3 is −21.1430 kcal/mol, its performance in sensory evaluation is not particularly outstanding. In contrast, the peptide LGPK, with a binding free energy of −10.2868 kcal/mol with T1R3, demonstrates stronger umami characteristics in sensory evaluation than DSWPSL. This suggests that the strength of binding energy does not necessarily correlate with the actual sensory effect, implying that high-affinity binding does not always lead to effective functional activation.

This discrepancy between binding and activation may be attributed to several factors.

1) Steric hindrance due to spatial structure: Some peptides, while capable of strong binding with the receptor, may have spatial structures that prevent optimal docking. For example, larger side chains may restrict the peptide from fully interacting with the receptor's active site, thereby limiting signal activation. The DSWPSL peptide, for instance, is unable to effectively access the NA1 region of T1R3 due to its large side chains, impairing signal activation.
2) Suboptimal binding geometry: While high-affinity binding generally indicates a stable complex, the geometry of the peptide-receptor binding may not always be ideal. The angle or distance of the interaction may not be optimal, affecting the efficiency of receptor signal transduction. Even strong binding can be ineffective if the structural match between the peptide and receptor is not ideal.
3) Functional differences between receptor subunits: Although both T1R1 and T1R3 can bind to umami peptides, their functional roles in activating the signaling pathways may differ. T1R3 may be more suitable for certain peptides, especially those with stronger hydrophilic or charged properties. Therefore, even peptides that exhibit strong binding may not effectively activate the signaling pathway, which in turn affects the sensory outcome.

This phenomenon highlights the difference between binding affinity and functional activation, underscoring the importance of considering not only the binding strength but also the structural compatibility of the peptide-receptor interaction and the ability to activate the receptor signaling pathway in future research. Enhancing binding affinity alone may not guarantee effective umami perception, providing more detailed guidance for the design and screening of umami peptides.

## 5 Conclusions

In this study, we successfully identified umami peptides from soybean proteins that bind to the T1R1 and T1R3 subunits of the umami receptor. Using four deep learning models, WLN, HGNN5, AttentiveFP, and GraphSAGE, we predicted the umami activity of these peptides. Peptides predicted as umami by at least two models were selected for further analysis, including virtual hydrolysis, biological activity, solubility, hemolysis, toxicity predictions, and molecular docking. Four peptides, DSWPSL, SHHPR, LGPK, and SSW, were identified as potential umami peptides. Electronic tongue experiments confirmed that all four peptides exhibited umami properties, with SHHPR exhibiting the most pronounced umami sensation.

Interestingly, the WLN model did not predict SHHPR as an umami peptide despite having the strongest umami effect in the experiments. To explore the binding mechanisms, 200 ns molecular dynamics simulations were performed. The results indicated that SHHPR and LGPK formed more stable complexes with T1R3 than with T1R1. Binding free energy calculations revealed that T1R3 had a stronger correlation with the experimental data than T1R1, suggesting that umami peptides are more likely to induce umami perception by binding to T1R3. Decomposition of binding free energy further supports this, with SHHPR showing stronger interactions with T1R3 than with T1R1 due to its C-terminus entering the NA1 region.

This study offers an innovative approach to screen umami peptides by integrating multiple prediction models with experimental validation. It also provides insights into the molecular mechanisms of umami perception, suggesting that future umami peptide design should avoid large hydrophobic groups near the N-terminus, particularly in positively charged regions, to enhance the binding affinity with T1R3.

## Sensory evaluation statement

The sensory evaluation experiment has been fully explained to all participants, and their informed consent has been obtained. All participants are aware of the purpose and process of the experiment, as well as the sensory criteria required for the scoring task. They understand that the results will be used for analysis in this study. Participants will independently and objectively score the samples during the evaluation process. The data collected will be strictly confidential and used solely for scientific research, ensuring the protection of participants' privacy.

## CRediT authorship contribution statement

**Xiaoli Shen:** Writing – original draft, Visualization, Validation, Software, Formal analysis, Data curation, Conceptualization. **Hao Zhang:** Writing – review & editing, Validation, Formal analysis, Conceptualization. **Pengyin Zhang:** Methodology, Investigation. **Xiaodi Niu:** Supervision, Investigation. **Xuerui Zhao:** Methodology, Investigation. **Lvzhou Zhu:** Methodology. **Jinyang Zhu:** Software. **Song Wang:** Supervision, Resources, Project administration, Funding acquisition.

## Funding sources

This research was funded by the Jilin Provincial Science and Technology Development Plan (grant number: 20220508114RC), the Jilin University Graduate Teaching Reform Project (grant number: 2022JGZ032), the Changchun MaTai Biotechnology Co., Ltd. Technology Development Project (grant number: 3R1210726449), and the Jilin Provincial Science and Technology Development Plan (grant number: YDZJ202501ZYTS299).

## Declaration of competing interest

The authors declare that they have no known competing financial interests or personal relationships that could have appeared to influence the work reported in this paper.

## Data Availability

The data that has been used is confidential.

## Appendix A Supplementary data

Supplementary material. To gain a comprehensive understanding of the results, please refer to the supplementary tables and figures, which can be found in Appendix A. Image 1
