# Supplementary material for: In silico analysis of soybean-derived umami peptides: Discovery and interaction mechanisms with T1R1/T1R3 receptor
Source: Food Chem X. 2025 May 10;28:102544. doi: 10.1016/j.fochx.2025.102544 (PMC12141843; doi:10.1016/j.fochx.2025.102544)
Supplement: Supplementary file 1 — Supplementary material. To gain a comprehensive understanding of the results, please refer to the supplementary tables and figures, which can be found in Appendix A. [file mmc1.docx]

Table S1. Peptides with biological activity.

| Peptide | Bioactive |
| --- | --- |
| CW | 0.99581 |
| MW | 0.995312 |
| GF | 0.994712 |
| CMW | 0.993837 |
| GW | 0.993164 |
| PW | 0.992911 |
| LF | 0.986934 |
| LMF | 0.979926 |
| RPF | 0.977766 |
| PFPY | 0.969077 |
| CPPSF | 0.96497 |
| WMA | 0.961852 |
| AGW | 0.959298 |
| MDF | 0.959246 |
| CPCR | 0.949732 |
| IF | 0.949173 |
| SF | 0.948796 |
| QF | 0.946135 |
| IW | 0.944175 |
| NF | 0.941145 |
| DGF | 0.939426 |
| HGF | 0.938552 |
| PDF | 0.934107 |
| SW | 0.93391 |
| DW | 0.933025 |
| QW | 0.928524 |
| HPIW | 0.896869 |
| ML | 0.894564 |
| GG | 0.88736 |
| LMP | 0.886515 |
| WPVL | 0.880808 |
| EGCW | 0.876556 |
| HPNF | 0.875719 |
| CR | 0.865233 |
| PGL | 0.855192 |
| ADPNF | 0.852384 |
| MR | 0.849148 |
| GGL | 0.838635 |
| CY | 0.831032 |
| MDSF | 0.829954 |
| EMW | 0.82038 |
| SHW | 0.816324 |
| VF | 0.815398 |
| NGDW | 0.815121 |
| TW | 0.814447 |
| GGR | 0.81127 |
| GL | 0.808777 |
| SIF | 0.805497 |
| MEW | 0.801147 |
| PR | 0.787626 |
| DSWPSL | 0.785783 |
| DNF | 0.775374 |
| ISW | 0.757343 |
| INPYPR | 0.756391 |
| QNCMY | 0.75044 |
| INF | 0.749347 |
| AM | 0.74549 |
| GY | 0.741592 |
| SSW | 0.731445 |
| CDGY | 0.719177 |
| RPY | 0.716662 |
| HGVF | 0.702486 |
| APAP | 0.70062 |
| QWME | 0.691206 |
| AMR | 0.688815 |
| SSAMM | 0.68653 |
| LDMGP | 0.684233 |
| SSIW | 0.68172 |
| LIGC | 0.676497 |
| DML | 0.667515 |
| LISW | 0.665913 |
| AANW | 0.66488 |
| LYPR | 0.663945 |
| CI | 0.660168 |
| LGL | 0.652338 |
| HSILMP | 0.649456 |
| DCY | 0.64835 |
| CPK | 0.648049 |
| MGD | 0.644133 |
| IGG | 0.642808 |
| AVW | 0.63114 |
| SC | 0.620334 |
| LL | 0.618551 |
| LHM | 0.614823 |
| NM | 0.604672 |
| TIGCAW | 0.601279 |
| NGMIR | 0.599087 |
| LHPGR | 0.594419 |
| GDMDY | 0.589397 |
| CSGHR | 0.589257 |
| EW | 0.585631 |
| SGNIPY | 0.580003 |
| IVF | 0.57343 |
| SVF | 0.573302 |
| LAC | 0.571138 |
| LR | 0.569984 |
| MD | 0.569594 |
| GLPK | 0.561671 |
| DVF | 0.551408 |
| DLP | 0.549727 |
| VDW | 0.548779 |
| AMQL | 0.545898 |
| NTW | 0.543895 |
| CQ | 0.540359 |
| AGY | 0.540291 |
| SHHPR | 0.538536 |
| RPIR | 0.532517 |
| IGR | 0.532161 |
| NGNC | 0.53085 |
| HPNL | 0.523186 |
| LGPK | 0.516678 |
| LY | 0.516639 |
| MTPR | 0.515171 |
| LQPY | 0.51421 |
| CS | 0.513886 |
| LAP | 0.512708 |
| ISCR | 0.504779 |
| SAC | 0.503776 |

Table S2. Peptides with good water solubility and low hemolytic activity.

| Sequence | Hemolytic | Soluble | Nonfouling | Synthesis |
| --- | --- | --- | --- | --- |
| SGNIPY | 0% | 97% | 82% | 1.22 |
| CPPSF | 0% | 94% | 79% | 1.21 |
| ADPNF | 0% | 91% | 82% | 1.1 |
| DSWPSL | 0% | 90% | 80% | 1.11 |
| CDGY | 0% | 67% | 72% | 1.08 |
| SSW | 1% | 98% | 65% | 1.07 |
| CS | 1% | 98% | 59% | 1 |
| SHW | 1% | 97% | 53% | 0.99 |
| SSAMM | 1% | 97% | 65% | 0.91 |
| AANW | 1% | 96% | 51% | 0.89 |
| GDMDY | 1% | 96% | 82% | 1.06 |
| HSILMP | 1% | 94% | 53% | 1 |
| APAP | 1% | 93% | 82% | 1.19 |
| AMQL | 1% | 91% | 51% | 0.76 |
| SHHPR | 1% | 91% | 83% | 1.15 |
| SF | 1% | 84% | 54% | 0.98 |
| AGY | 1% | 76% | 64% | 1.07 |
| INPYPR | 1% | 70% | 82% | 1.2 |
| GGL | 1% | 68% | 86% | 1.23 |
| GG | 1% | 64% | 91% | 1.47 |
| ISCR | 1% | 63% | 55% | 0.93 |
| GL | 1% | 61% | 67% | 1.11 |
| SC | 2% | 98% | 59% | 1 |
| SAC | 2% | 97% | 54% | 0.92 |
| AM | 2% | 86% | 51% | 0.72 |
| EGCW | 2% | 85% | 60% | 0.97 |
| CSGHR | 2% | 81% | 77% | 1.08 |
| CPK | 3% | 94% | 83% | 1.11 |
| GLPK | 3% | 93% | 92% | 1.19 |
| SSIW | 4% | 98% | 49% | 0.99 |
| AGW | 4% | 82% | 56% | 1 |
| GW | 4% | 80% | 59% | 1.13 |
| TW | 4% | 78% | 40% | 0.89 |
| AVW | 4% | 63% | 30% | 0.76 |
| TIGCAW | 4% | 61% | 47% | 0.92 |
| SW | 5% | 98% | 51% | 1 |
| LGPK | 5% | 72% | 92% | 1.19 |
| CMW | 7% | 83% | 35% | 0.76 |
| QW | 8% | 94% | 53% | 0.81 |
| SIF | 9% | 68% | 35% | 0.9 |
| PW | 9% | 66% | 60% | 1.21 |
| GF | 9% | 64% | 61% | 1.11 |
| CY | 10% | 97% | 45% | 0.89 |

Table S3. The results of predicting peptide taste using HGNN, WLN, AttentiveFP, and GraphSAGE.

| Number | Peptide | HGNN | WLN | AttentiveFP | GraphSAGE |
| --- | --- | --- | --- | --- | --- |
| 1 | SGNIPY | Astringent | Astringent | Astringent | Astringent |
| 2 | CPPSF | Kokumi | Kokumi | Kokumi | Umami |
| 3 | ADPNF | Astringent | Astringent | Astringent | Astringent |
| 4 | DSWPSL | Umami | Umami | Kokumi | Umami |
| 5 | CDGY | Kokumi | Umami | Kokumi | Astringent |
| 6 | SSW | Umami | Umami | Astringent | Umami |
| 7 | CS | Sweet | Umami | Umami | Umami |
| 8 | SHW | Astringent | Astringent | Astringent | Astringent |
| 9 | SSAMM | Umami | Umami | Astringent | Umami |
| 10 | AANW | Umami | Astringent | Astringent | Astringent |
| 11 | GDMDY | Astringent | Umami | Umami | Umami |
| 12 | HSILMP | Umami | Kokumi | Kokumi | Umami |
| 13 | APAP | Umami | Umami | Umami | Astringent |
| 14 | AMQL | Umami | Umami | Astringent | Umami |
| 15 | SHHPR | Umami | Kokumi | Umami | Umami |
| 16 | SF | Bitter | Astringent | Astringent | Umami |
| 17 | AGY | Umami | Astringent | Astringent | Astringent |
| 18 | INPYPR | Kokumi | Kokumi | Kokumi | Umami |
| 19 | GGL | Umami | Astringent | Umami | Umami |
| 20 | GG | Salty | Kokumi | Tasteless | Astringent |
| 21 | ISCR | Astringent | Astringent | Kokumi | Astringent |
| 22 | GL | Umami | Umami | Umami | Umami |
| 23 | SC | Sweet | Umami | Umami | Umami |
| 24 | SAC | Astringent | Umami | Umami | Umami |
| 25 | AM | Astringent | Umami | Umami | Umami |
| 26 | EGCW | Kokumi | Umami | Kokumi | Astringent |
| 27 | CSGHR | Umami | Kokumi | Kokumi | Umami |
| 28 | CPK | Umami | Umami | Kokumi | Umami |
| 29 | GLPK | Umami | Umami | Kokumi | Umami |
| 30 | SSIW | Astringent | Astringent | Umami | Astringent |
| 31 | AGW | Astringent | Astringent | Astringent | Astringent |
| 32 | GW | Astringent | Bitter | Astringent | Astringent |
| 33 | TW | Umami | Astringent | Astringent | Astringent |
| 34 | AVW | Umami | Astringent | Umami | Astringent |
| 35 | TIGCAW | Astringent | Astringent | Astringent | Astringent |
| 36 | SW | Umami | Umami | Astringent | Umami |
| 37 | LGPK | Umami | Umami | Kokumi | Umami |
| 38 | CMW | Kokumi | Kokumi | Kokumi | Umami |
| 39 | QW | Kokumi | Umami | Astringent | Astringent |
| 40 | SIF | Astringent | Astringent | Umami | Astringent |
| 41 | PW | Kokumi | Umami | Bitter | Umami |
| 42 | GF | Astringent | Astringent | Astringent | Astringent |
| 43 | CY | Astringent | Kokumi | Astringent | Astringent |

Table S4. The ΔG_elec_ contributions of important residues in the T1R1-peptide system (kcal/mol).

|  | T1R1-DSWPSL | T1R1-SHHPR | T1R1-LGPK | T1R1-SSW |
| --- | --- | --- | --- | --- |
| Met1 | -3.1007 | 3.3356 | 3.9431 | 0.3839 |
| Arg7 | -3.2443 | 3.9329 | 4.078 | 0.1543 |
| Glu27 | 5.2836 | -5.5553 | -5.2043 | 0.1596 |
| Asp31 | 5.0804 | -6.3469 | -5.8409 | 0.7316 |
| Asp37 | 3.7328 | -4.3821 | -4.3848 | 0.0592 |
| Arg54 | -14.5651 | 6.7623 | 10.1322 | 0.7476 |
| Arg56 | -7.9003 | 6.6032 | 5.9552 | 0.3833 |
| Glu58 | 8.6165 | -7.3546 | -6.6069 | -0.5221 |
| Asp63 | 7.6653 | -7.1669 | -7.581 | 0.8421 |
| Arg64 | -12.9431 | -15.3552 | 10.5823 | -1.3116 |
| Glu70 | 4.4561 | -13.2863 | -7.575 | 5.6723 |
| Arg80 | -5.1776 | 6.7265 | 6.3402 | -0.8855 |
| Glu84 | 4.0205 | -5.0471 | -4.9895 | 0.4591 |
| Glu85 | 4.0519 | -5.0154 | -5.171 | 0.5065 |
| Asp104 | 9.8716 | -11.143 | -9.0949 | 1.4994 |
| Asp108 | 13.2402 | -26.4404 | -11.9435 | -3.5992 |
| Arg117 | -5.8354 | 6.6032 | 6.1896 | 0.2322 |
| Glu128 | 4.1566 | -4.4828 | -4.4323 | -0.284 |
| Asp132 | 3.8574 | -4.3603 | -4.5229 | -0.3391 |
| Asp147 | 12.8453 | -24.6416 | -24.3789 | -9.8022 |
| Arg151 | -15.2936 | 22.8664 | 10.7209 | 3.9786 |
| Glu174 | 5.9897 | -7.546 | -9.3676 | -1.3608 |
| Lys179 | -5.6655 | 7.0574 | 7.3264 | 1.0502 |
| Arg180 | -4.7554 | 5.5768 | 5.9207 | 0.9181 |
| Arg187 | -6.8582 | 9.7279 | 13.1062 | 1.6135 |
| Asp192 | 7.3055 | -10.5981 | -18.5414 | -2.5332 |
| Lys193 | -5.4716 | 7.0714 | 8.4096 | 1.0083 |
| Glu197 | 4.9537 | -6.1102 | -7.1371 | -0.3836 |
| Lys205 | -4.272 | 4.9879 | 5.2564 | -0.2023 |
| Asp218 | 12.9051 | -28.9935 | -17.4318 | -18.4904 |
| Asp219 | 9.3013 | -18.3167 | -16.9929 | -12.9787 |
| Glu229 | 5.6724 | -6.9139 | -6.9106 | -0.7972 |
| Lys242 | -6.2426 | 7.5061 | 7.5334 | 0.9674 |
| Asp243 | 8.284 | -9.2567 | -8.1686 | -0.9112 |
| Asp253 | 12.3271 | -30.9161 | -7.8658 | -0.2703 |
| Glu254 | 7.9506 | -9.1721 | -5.9315 | -0.0942 |
| Arg255 | -11.9882 | 10.8256 | 8.2277 | 0.7244 |
| Arg261 | -7.1528 | 7.4885 | 5.4917 | 0.1032 |
| Arg277 | -32.9888 | 18.9323 | 5.913 | -26.398 |
| Arg281 | -16.4442 | 12.1827 | 8.513 | -3.573 |
| Glu285 | 10.6728 | -9.737 | -7.3148 | 1.8153 |
| Lys295 | -5.9865 | 6.5403 | 5.6608 | -0.1699 |
| Glu301 | 9.435 | -13.6551 | -21.922 | 2.3697 |
| Arg307 | -6.5282 | 9.383 | 6.4665 | -3.1136 |
| His308 | -5.1834 | -0.0557 | 0.576 | -0.7989 |
| Arg317 | -6.6252 | 6.3254 | 5.1424 | -0.6742 |
| Lys328 | -4.8632 | 6.244 | 7.6744 | -3.2645 |
| Arg329 | -4.7148 | 6.144 | 6.491 | -0.6799 |
| Lys335 | -4.2428 | 5.762 | 5.0502 | -1.0446 |
| Glu338 | 4.8621 | -6.7487 | -6.0507 | 1.3358 |
| Glu339 | 4.0067 | -5.2728 | -4.6327 | 0.7204 |
| Arg343 | -3.8893 | 4.873 | 4.4847 | -0.5346 |
| Asp345 | 4.081 | -4.7004 | -4.3871 | 0.4213 |
| Lys346 | -4.7931 | 5.7103 | 5.2397 | -0.5095 |
| Lys347 | -4.3162 | 4.5494 | 4.1851 | -0.2803 |
| Arg350 | -4.7873 | 4.8967 | 4.4343 | -0.3307 |
| Lys354 | -6.0164 | 5.2907 | 3.878 | -0.2307 |
| Arg365 | -6.9366 | 7.0247 | 6.4318 | -1.0079 |
| Glu366 | 5.6908 | -8.009 | -6.5807 | 0.6455 |
| Lys377 | -4.8821 | 6.3266 | 5.4969 | -1.1279 |
| Lys379 | -5.6742 | 6.9632 | 4.7344 | -4.0409 |
| Arg391 | -5.231 | 6.9769 | 8.4919 | -0.532 |
| Arg413 | -3.4248 | 3.7015 | 3.7556 | 0.0299 |
| Arg415 | -3.7443 | 4.4121 | 4.4941 | 0.1258 |
| Glu423 | 4.0415 | -4.8161 | -5.3977 | -0.3514 |
| Lys427 | -3.7283 | 4.6375 | 5.14 | 0.2169 |
| Lys434 | -3.6107 | 4.4725 | 4.8416 | -0.408 |
| Asp435 | 4.5824 | -5.9308 | -6.6367 | 0.483 |
| Asp441 | 4.2048 | -5.2666 | -6.3056 | -0.3477 |
| Arg443 | -4.6179 | 5.8818 | 6.6496 | 0.5346 |
| Asp444 | 5.7212 | -7.5316 | -9.5751 | -0.8499 |
| Asp455 | 5.1435 | -5.7078 | -5.249 | 0.8353 |
| Lys460 | -5.4441 | 5.9108 | 5.0566 | -0.9284 |
| Glu480 | 4.0909 | -4.6339 | -4.6172 | 0.2788 |
| Lys482 | -4.2914 | 5.0553 | 4.9565 | -0.4834 |
| Lys488 | -4.1879 | 4.4954 | 4.1039 | -0.2908 |
| Asp489 | 4.2748 | -4.4557 | -4.281 | 0.3358 |
| Asn490 | 4.1775 | -4.6424 | -4.1507 | 0.366 |

Table S5. The ΔG_elec_ contributions of important residues in the T1R3-peptide system (kcal/mol).

|  | T1R3-DSWPSL | T1R3-SHHPR | T1R3-LGPK | T1R3-SSW |
| --- | --- | --- | --- | --- |
| Met1 | -3.5899 | 3.5666 | 3.367 | 0.0376 |
| Arg30 | -4.9114 | 5.1958 | 4.747 | -0.7895 |
| Lys32 | -4.0519 | 4.327 | 4.3351 | -0.5797 |
| Asp34 | 3.9675 | -3.998 | -4.5784 | 0.2707 |
| Glu45 | -2.9754 | -24.672 | -5.2082 | 2.4012 |
| Glu47 | 10.8682 | -12.9476 | -4.7399 | 2.2975 |
| Glu48 | 13.9356 | -19.7893 | -4.3682 | 1.9361 |
| Arg52 | -12.5906 | 11.8985 | 4.2576 | -1.5891 |
| Arg54 | -27.3385 | 11.7601 | 4.1027 | -5.229 |
| Arg56 | -11.0636 | 8.0676 | 4.2976 | -2.2457 |
| Arg64 | -7.6528 | 14.7638 | 5.1851 | -1.8068 |
| Asn68 | -5.519 | 0.8697 | -1.9509 | 0.1228 |
| Lys77 | -4.8785 | 5.5535 | 6.6225 | -0.0182 |
| Glu81 | 4.5487 | -4.9195 | -6.3329 | -0.053 |
| Glu82 | 4.3904 | -4.7718 | -7.689 | -0.5688 |
| Lys86 | -4.1651 | 4.5173 | 7.1157 | 0.4983 |
| Asp88 | 3.7709 | -4.1395 | -6.0548 | -0.3587 |
| Arg94 | -3.9554 | 4.0845 | 4.979 | -0.1152 |
| Asp98 | 5.6509 | -5.5941 | -5.3139 | 1.3133 |
| Asp101 | 7.5709 | -11.2851 | -5.6842 | 5.0341 |
| Ser104 | -2.0155 | -4.4881 | 0.1798 | -5.0917 |
| Glu105 | 7.6158 | -28.8649 | -4.8047 | 9.1256 |
| Lys111 | -9.3714 | 8.8155 | 4.4371 | -2.8867 |
| Lys119 | -4.3872 | 4.1079 | 3.5161 | -0.6963 |
| Arg123 | -5.1096 | 5.0365 | 3.7562 | -1.0375 |
| Asp124 | 5.1961 | -4.9315 | -3.6048 | 1.0512 |
| Arg137 | -5.3989 | 5.4146 | 4.3182 | -1.1726 |
| His145 | -3.317 | 3.3493 | 0.3742 | -5.8024 |
| Ser146 | -1.5402 | -10.5447 | -1.6745 | -3.9865 |
| Ser147 | -0.4434 | -14.1377 | -1.5841 | -0.8373 |
| Glu148 | 18.5326 | -16.7274 | -5.1271 | 10.5843 |
| Lys155 | -9.4396 | 6.6165 | 5.0831 | -2.1544 |
| Ala169 | -0.2116 | -5.8973 | -0.9432 | -0.5746 |
| Glu172 | 6.9224 | -7.151 | -9.0145 | 0.112 |
| Arg177 | -6.2486 | 5.2467 | 6.8605 | -0.5728 |
| Glu178 | 6.0318 | -5.1314 | -4.9591 | 0.6698 |
| Arg185 | -8.1253 | 5.4159 | 11.5046 | -0.4066 |
| Asp190 | 8.0732 | -25.8681 | -21.1144 | -1.8856 |
| Arg191 | -6.4326 | 7.4454 | 9.0905 | 0.3087 |
| Glu199 | 4.8815 | -6.1653 | -7.2776 | -0.9782 |
| Glu203 | 4.3379 | -5.3437 | -6.7476 | -1.0401 |
| Asp215 | 13.837 | -10.2924 | -5.627 | 1.6525 |
| Asp216 | 15.0623 | -17.547 | -6.2604 | 2.1115 |
| Glu217 | 11.8255 | -9.0181 | -5.632 | 4.4049 |
| Arg220 | -13.9882 | 9.8222 | 5.4402 | -2.2414 |
| Arg233 | -5.2805 | 6.2712 | 7.2077 | 0.8385 |
| Glu240 | 6.8717 | -6.7413 | -5.7018 | -0.0862 |
| Arg247 | -42.0712 | 14.1409 | 4.8934 | -3.7529 |
| Asp249 | 13.2982 | -9.136 | -4.3902 | 1.1709 |
| Asp250 | 10.0193 | -7.427 | -4.4292 | 0.5211 |
| Arg252 | -19.685 | 11.8719 | 4.8569 | -2.3349 |
| Lys255 | -9.9806 | 7.8169 | 4.6941 | -0.5452 |
| Asp258 | 7.1419 | -6.1742 | -4.6294 | 0.013 |
| Arg291 | -5.3255 | 5.3185 | 5.1469 | 0.8661 |
| Lys295 | -5.6946 | 5.9608 | 5.9849 | 1.0389 |
| Glu301 | 12.4413 | -32.5191 | -37.05 | -23.6511 |
| Ala302 | 0.8128 | 0.5989 | 0.0831 | -6.5617 |
| Asp307 | 3.2847 | -9.8073 | -16.6467 | -8.0932 |
| Arg327 | -5.2169 | 7.3471 | 11.9809 | 1.7948 |
| Glu333 | 4.1923 | -5.0247 | -8.8047 | -0.9379 |
| Lys339 | -4.1096 | 5.2051 | 7.884 | 0.9097 |
| Asp347 | 3.7555 | -4.4149 | -4.613 | 0.0178 |
| Glu356 | 3.8498 | -4.1941 | -3.6464 | 0.3426 |
| Arg357 | -3.8602 | 3.9801 | 4.2685 | -0.2668 |
| Glu358 | 4.2057 | -4.829 | -4.5343 | 0.5223 |
| Glu362 | 5.4573 | -5.5562 | -4.5156 | 1.1605 |
| Glu363 | 5.6137 | -6.124 | -4.1749 | 1.3308 |
| Asp364 | 5.45 | -5.464 | -3.8033 | 1.1842 |
| Arg369 | -5.4483 | 5.7209 | 4.9683 | -1.1291 |
| Asp374 | 5.2058 | -6.5098 | -4.8564 | 0.7437 |
| His387 | -4.9808 | 0.7245 | -0.3584 | 3.6758 |
| Asp419 | 3.7482 | -3.4738 | -3.8998 | 0.285 |
| Lys422 | -4.2227 | 3.966 | 4.0839 | -0.5178 |
| Glu428 | 4.4383 | -4.2375 | -4.9796 | 0.2901 |
| Arg443 | -4.5955 | 5.0798 | 7.5637 | 0.3538 |
| Asp445 | 5.5125 | -6.2477 | -7.8731 | -0.2367 |
| Asp451 | 5.5072 | -7.0153 | -9.5599 | -0.9141 |
| Glu453 | 5.5195 | -7.4008 | -11.641 | -1.6974 |
| Asp455 | 6.504 | -8.95 | -16.0987 | -3.192 |
| Lys457 | -6.8169 | 9.0921 | 19.6263 | 4.0838 |
| Arg467 | -5.1488 | 6.453 | 14.0306 | 2.644 |
| Asp470 | 5.6555 | -7.2598 | -14.5815 | -2.8186 |
| Arg473 | -5.4799 | 6.7068 | 12.6312 | 2.1012 |
| Arg479 | -4.2381 | 5.3492 | 8.109 | 1.169 |
| Glu481 | 4.3841 | -5.5023 | -7.7955 | -1.4021 |
| Arg482 | -4.2944 | 5.1601 | 6.5182 | 1.0936 |
| Lys484 | -4.544 | 5.319 | 8.1245 | 1.5956 |
| Arg486 | -4.3375 | 5.198 | 7.5172 | 1.5142 |
| Ser490 | 4.2162 | -4.5722 | -5.7649 | -0.999 |


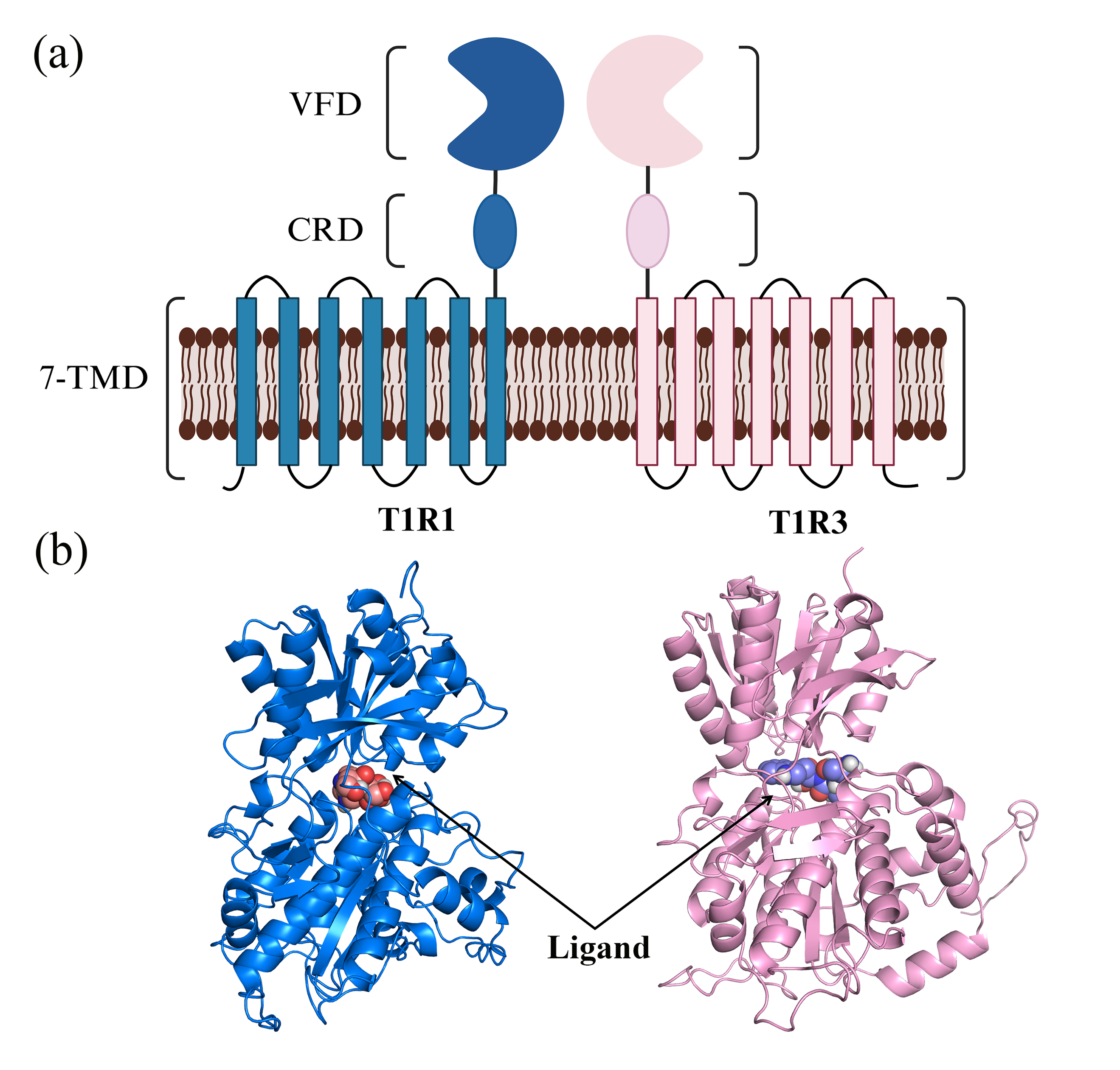


Figure S1. (a) The structure of the umami receptor T1R1/T1R3 and (b) its binding cavities. Figure a was created in BioRender. Shen, X. (2024) https://BioRender.com/x40p315.


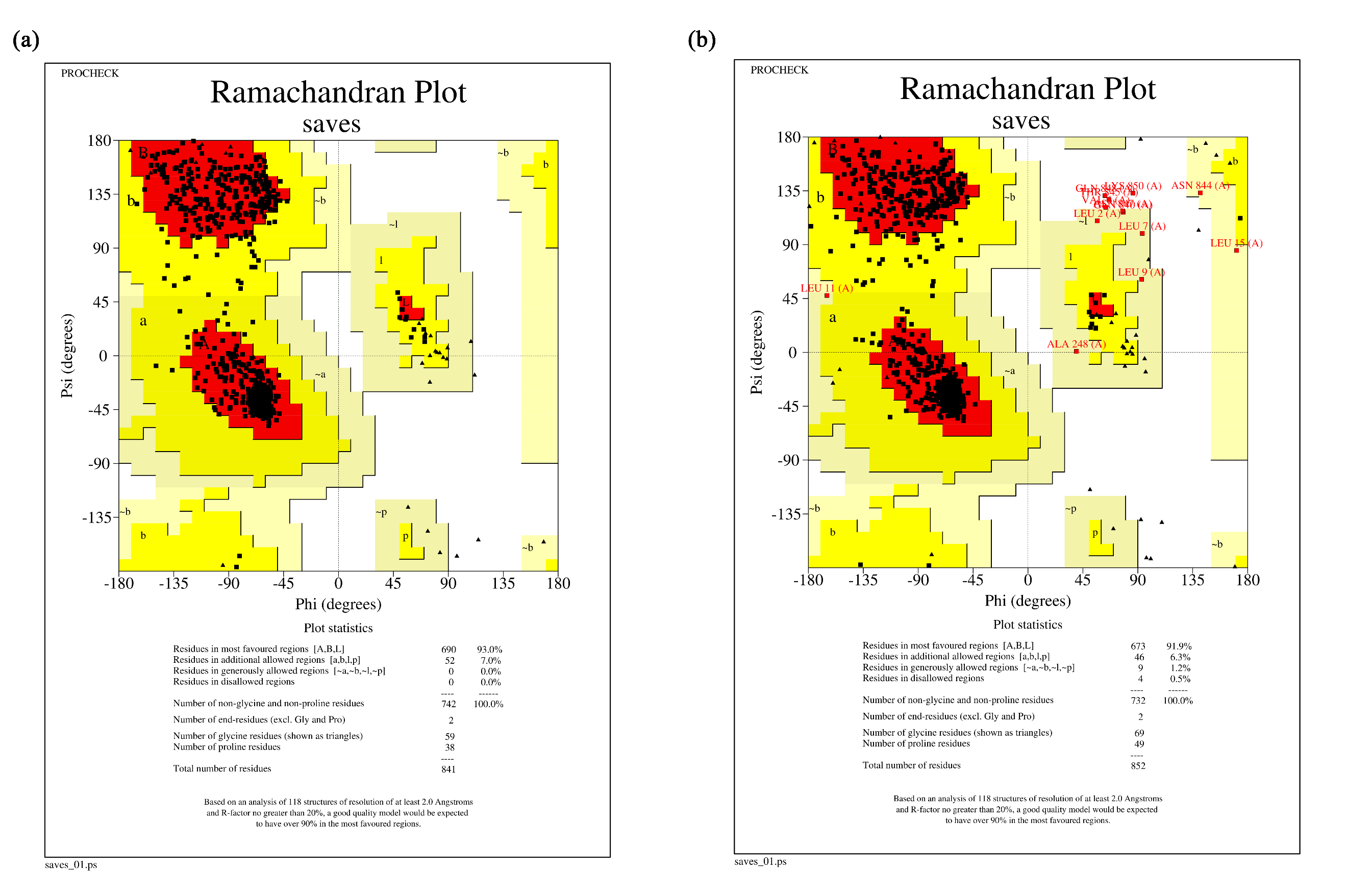


Figure S2. The Ramachandran plot validation of the receptor structures. (a) T1R1. (b) T1R3.


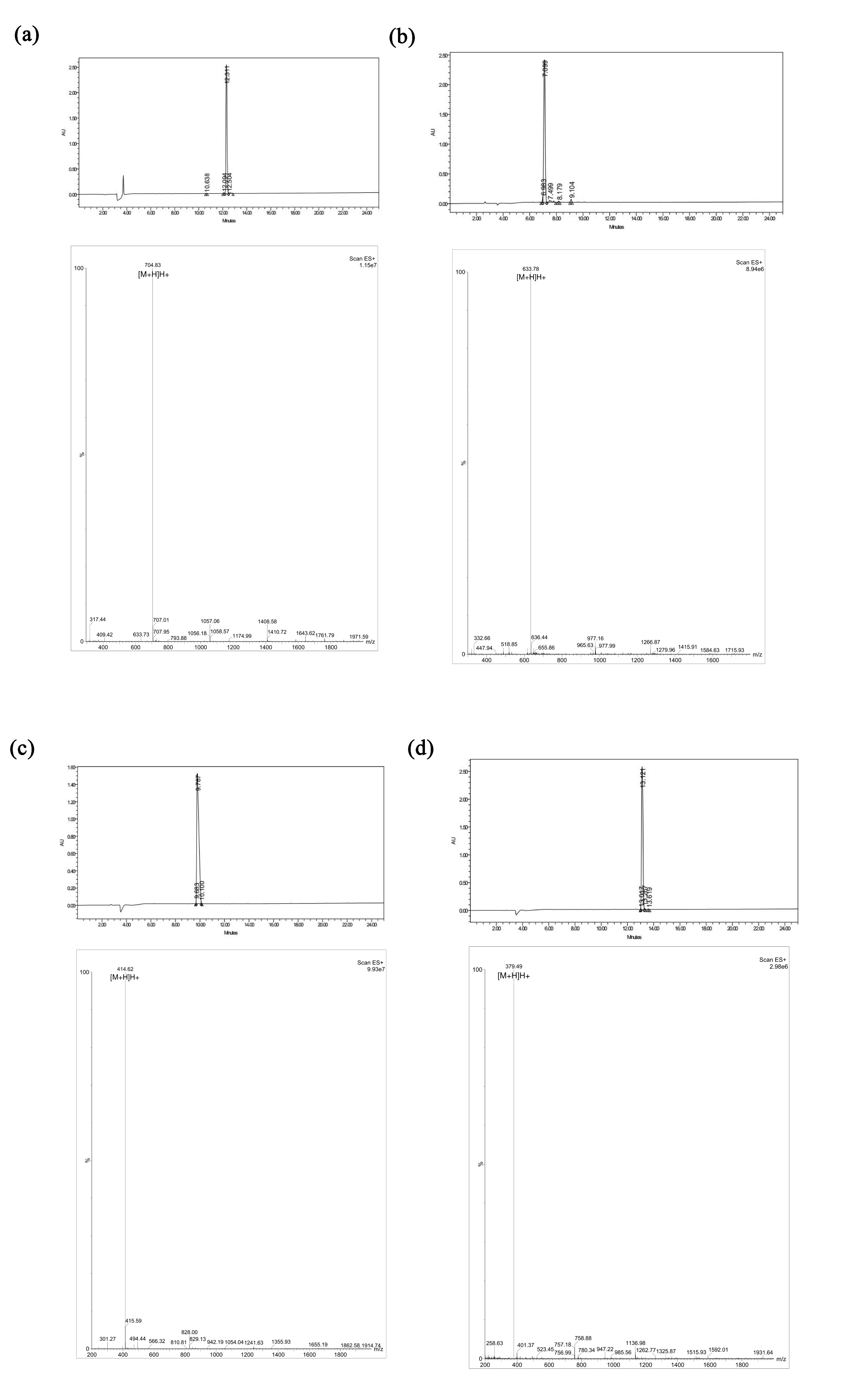


Figure S3. The results of HPLC-MS. (a) DSWPSL. (b) SHHPR. (c) LGPK. (d) SSW.


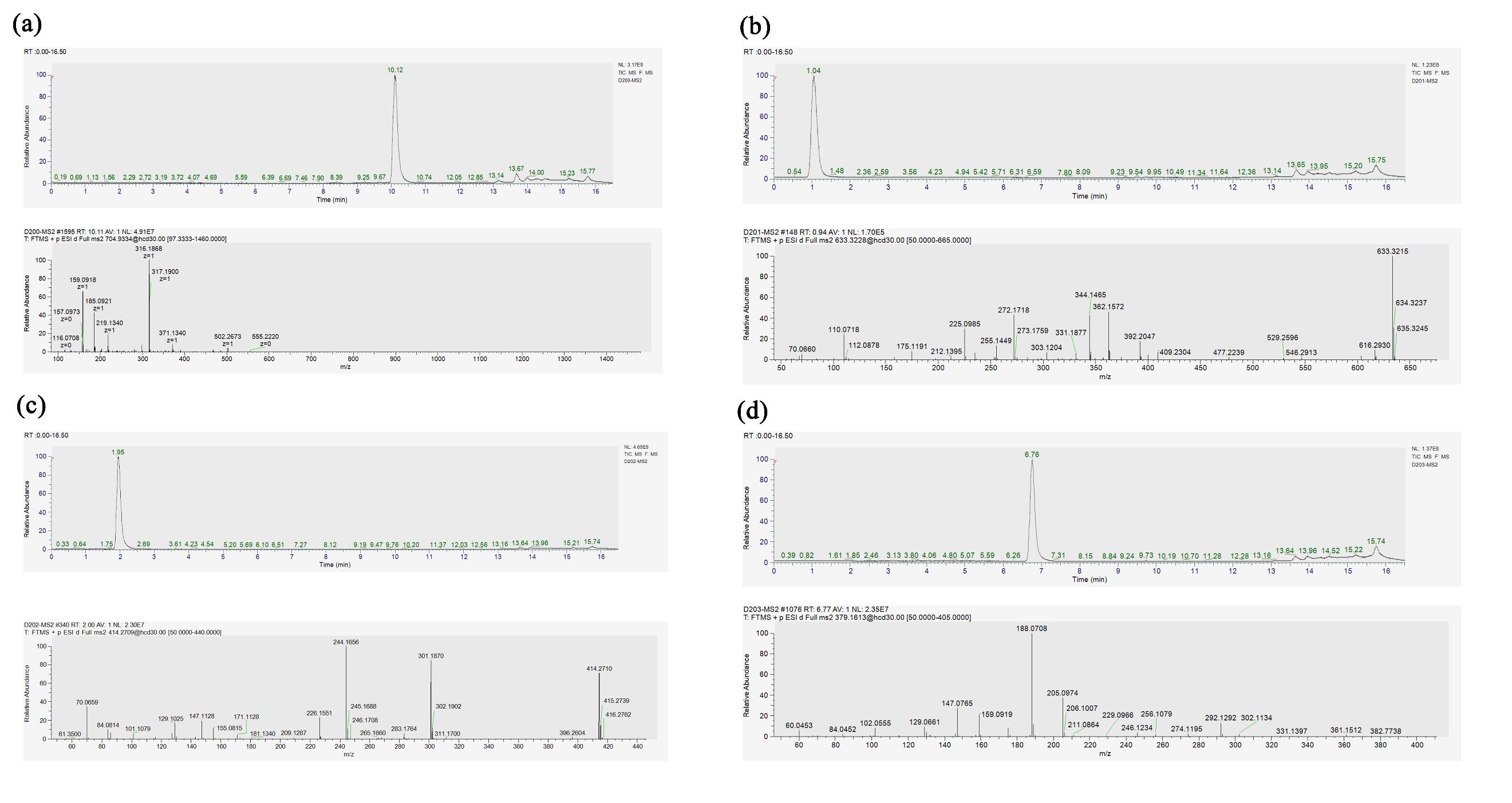


Figure S4. The results of TIC and MS/MS analysis. The TIC plots are shown on top, and the MS/MS spectra are shown below. (a) DSWPSL. (b) SHHPR. (c) LGPK. (d) SSW.

**Peptide Synthesis and** **HPLC-MS, TIC and MS/MS Analysis Procedures**

**1. Peptide Synthesis**

The peptides synthesized in this experiment were prepared using the solid-phase peptide synthesis (SPPS) method. The specific synthesis steps are as follows.

***1.1 Resin Selection and Pre-treatment***

**1.1.1 Resin Selection**

Choose an appropriate Fmoc-protected resin based on the C-terminal amino acid of the target peptide. For example:

DSWPSL: Use Fmoc-Leu-OH resin (C-terminal is leucine).

SHHPR: Use Fmoc-Arg (pbf)-OH resin (C-terminal is arginine).

LGPK: Use Fmoc-Lys (Boc)-OH resin (C-terminal is lysine).

SSW: Use Fmoc-Trp (Boc)-OH resin (C-terminal is tryptophan).

**1.1.2 Resin Pre-treatment**

Prior to synthesis, the resin needs to be pre-treated, which typically includes:

Cleaning the resin surface with dichloromethane (DCM) or DMF to remove impurities.

Drying the resin under a stream of nitrogen to ensure that the active groups on the resin are not contaminated.

***1.2 Synthesis Steps***

Each amino acid coupling step follows these standard procedures to ensure efficient attachment. The specific operations for each step are as follows:

**1.2.1 Deprotection of Fmoc Group**

The Fmoc protecting group is removed using a 20% piperidine/DMF solution:

Immerse the resin in a 20% piperidine/DMF solution and stir for 20 minutes.

After deprotection, wash the resin with DMF at least three times to remove unreacted piperidine.

**1.2.2 Amino Acid Coupling Reaction**

After deprotection, add the amino acid for coupling:

Prepare an amino acid solution using HBTU/NMM reagents to facilitate the reaction.

Mix HBTU and NMM in a 1:1 ratio, then add it to the resin for reaction with the amino acid.

**1.2.3 Amino Acid Coupling Verification**

After each amino acid coupling, verify the reaction using ninhydrin reagent:

Treat a small sample of the resin with a ninhydrin solution and observe any color changes.

A blue color indicates that the amino acid has been successfully coupled; if no change is observed, the reaction conditions need to be checked.

**1.2.4 Repeat the Process**

Following the target peptide sequence, repeat the deprotection, coupling, washing, and verification steps for each amino acid until the target peptide is fully synthesized.

***1.3 Specific Peptide Synthesis***

**i. DSWPSL (Asp-Ser-Trp-Pro-Ser-Leu)**

**Resin**: Use Fmoc-Leu-OH resin.

**Amino Acid Sequence**: Leu → Ser → Pro → Trp → Ser → Asp.

**Steps**:

Leu: Use Fmoc-Leu-OH resin to start, then proceed to the next step.

Ser: Use Fmoc-Ser (tbu)-OH, remove the Fmoc group, and add HBTU/NMM for a 40-minute reaction.

Pro: Use Fmoc-Pro-OH, deprotect the Fmoc group, then add the amino acid and wash the resin.

Trp: Use Fmoc-Trp (Boc)-OH, carefully remove the Boc protecting group and proceed as usual.

Ser: Repeat the Ser coupling.

Asp: Use Fmoc-Asp (OtBu)-OH, deprotect the Fmoc group, and couple the final amino acid.

**ii. SHHPR (Ser-His-His-Pro-Arg)**

**Resin**: Use Fmoc-Arg (pbf)-OH resin.

**Amino Acid Sequence**: Arg → Pro → His → His → Ser.

**Steps**:

Arg: Use Fmoc-Arg (pbf)-OH resin, deprotect the Fmoc group, then react with HBTU/NMM.

Pro: Use Fmoc-Pro-OH, remove the protecting group, and couple.

His: Use Fmoc-His (Trt)-OH, increase deprotection time to 40 minutes to ensure complete reaction.

His: Repeat the previous step for the second histidine residue.

Ser: Use Fmoc-Ser (tbu)-OH, deprotect the Fmoc group and couple.

**iii. LGPK (Leu-Gly-Pro-Lys)**

**Resin**: Use Fmoc-Lys (Boc)-OH resin.

**Amino Acid Sequence**: Lys → Pro → Gly → Leu.

**Steps**:

Lys: Use Fmoc-Lys (Boc)-OH resin, deprotect the Fmoc group, and proceed with the coupling.

Pro: Use Fmoc-Pro-OH, follow the standard procedure for coupling.

Gly: Use Fmoc-Gly-OH (note that glycine has no protecting group on its side chain).

Leu: Use Fmoc-Leu-OH to complete the peptide chain.

**iv. SSW (Ser-Ser-Trp)**

**Resin**: Use Fmoc-Trp (Boc)-OH resin.

**Amino Acid Sequence**: Trp → Ser → Ser.

**Steps**:

Trp: Use Fmoc-Trp (Boc)-OH resin, deprotect the Fmoc group, and add the amino acid.

Ser: Use Fmoc-Ser (tbu)-OH, repeat twice to complete the Ser coupling.

***1.4 Special Considerations***

i. Avoiding Side Reactions: Some amino acids, such as lysine and histidine, may undergo side reactions. Adjust the deprotection time or reaction conditions to minimize these effects.

ii. Poorly Soluble Peptides: For peptides with poor solubility, adding a small amount of DMSO or adjusting the reaction volume may help improve solubility.

iii. Resin Cleaning: After each synthesis, thoroughly clean the resin to avoid contamination in subsequent syntheses.

**2.** **HPLC-MS Analysis**

In this study, High-Performance Liquid Chromatography (HPLC) coupled with Mass Spectrometry (MS) was employed to analyze four synthesized peptides (DSWPSL, SHHPR, LGPK, and SSW). This method enables efficient separation and precise confirmation of the molecular weight of the peptides, ensuring their purity and structural integrity. The specific steps of the analysis are outlined below:

***2.1 Preparation of HPLC System***

**2.1.1 Instrument Setup**

The analysis was performed using an HPLC system equipped with a UV detector and C18 reverse-phase column for peptide separation.

i. Mobile Phase Composition:

Solvent A: 0.1% Trifluoroacetic acid (TFA) in water.

Solvent B: 0.1% TFA in acetonitrile.

ii. The flow Rate: Set at 1.0 mL/min to ensure optimal separation.

iii. Column Temperature: Maintained at 30°C to ensure reproducibility of the results.

iv. UV Detection Wavelength: Set to 214 nm, which is commonly used for peptide detection.

**2.1.2 Sample Preparation**

Each peptide (DSWPSL, SHHPR, LGPK, and SSW) was dissolved in an appropriate solvent (e.g., 0.1% TFA in water or 50% acetonitrile solution), and the concentration was adjusted to 1-2 mg/mL. A 20 µL aliquot of each peptide sample was injected for analysis.

**2.2 HPLC Separation Process**

i. Sample Injection
The prepared peptide samples (20 µL) were injected into the HPLC system for separation.

ii. Gradient Elution
The peptides were separated using gradient elution with solvents A and B as follows: Initially, the concentration of solvent B was set to 5%.

Over time, the acetonitrile concentration gradually increased to 50% and then further increased to 80%-90%.

iii. Gradient Example:

0-5 minutes: Solvent B 5%;

5-15 minutes: Solvent B increased from 5% to 50%;

15-25 minutes: Solvent B increased from 50% to 80%;

25-30 minutes: Solvent B increased to 90%;

This gradient condition effectively separated peptides based on their hydrophobicity.

iv. Separation Process
The peptides were separated based on their hydrophobicity during the gradient elution. Each peptide was separated in its respective retention time, depending on its chemical properties, ensuring effective and optimal separation.

**2.3 MS Analysis Process**

i. Mass Spectrometer Setup

Instrument Model: The analysis was conducted using the SHIMADZU LCMS-2020 mass spectrometer.

Ionization Mode: Electrospray Ionization (ESI) was used for sample ionization.

Nebulizer Gas Flow: Set at 1.5 L/min to ensure efficient ionization.

Probe Bias: Set to +4.5 kV to enhance ion formation efficiency.

CDL Temperature: Set to 250°C to ensure efficient ion transmission.

Detector Bias: Set to 1.2 kV for optimal ion detection.

Flow Rate: Set to 0.2 mL/min for optimal ion transmission.

ii. Sample Analysis
The samples separated by HPLC were introduced into the mass spectrometer for analysis. The peptides were ionized by electrospray ionization (ESI), and the resulting ions were analyzed for mass-to-charge ratios to confirm their molecular weight.

iii. Data Acquisition and Analysis
The molecular weight of each peptide was confirmed by analyzing the mass spectrum, focusing on the molecular ion peaks. The observed m/z values were compared to the theoretical molecular weights of the peptides. This analysis confirms the identity and purity of the synthesized peptides.

**2.4 Data Processing and Results Analysis**

1. HPLC Data Analysis
The chromatogram recorded by the UV detector was used to assess the purity of each peptide. Ideally, each peptide should appear as a single, sharp peak at its retention time, indicating high purity. The area under the main peak in the chromatogram was used to calculate the purity, which should be greater than 95% for each peptide.

2. MS Data Analysis
The mass spectrometry data were used to determine the molecular weight of each peptide. The observed molecular weight should match the theoretical value. The MS/MS data were also analyzed to confirm the structural integrity of the peptides and ensure that there were no degradation products or impurities.

**3. TIC and MS/MS Analysis**

Peptide analysis was performed using an Ultimate 3000 system coupled with a Q-Exactive mass spectrometer. Chromatographic separation was carried out using a C18 column (0.1 mm × 150 mm, 2.5 μm, Thermo), with a flow rate of 0.08 mL/min. The mobile phase A consisted of 0.1% formic acid (FA) in 98% water and 2% acetonitrile (ACN), while mobile phase B was 0.1% FA in 100% acetonitrile. A linear gradient was applied as follows: 1% B for the first 1 minute, followed by a linear increase to 60% B over the next 11 minutes, then to 95% B for 1 minute, and finally back to 2% B for the last 1 minute.

Mass spectrometry was conducted in ion mode with a top 5 data-dependent acquisition (DDA) scan. The detailed parameters were as follows: capillary temperature at 320°C; mass range from m/z 300 to 1000; AGC target of 1 × 10^6 for MS1 and 1 × 10^5 for MS2; source voltage at 3.3 kV. Data-dependent acquisition was used to trigger precursor isolation and sequencing, with precursor ions isolated within a 1.6 m/z window and fragmented by high-energy collision dissociation (HCD) at a collision energy of 30%.
